# Reactive navigation in extremely dense and highly intricate environments

**DOI:** 10.1371/journal.pone.0189008

**Published:** 2017-12-29

**Authors:** Javier Antich Tobaruela, Alberto Ortiz Rodríguez

**Affiliations:** Department of Mathematics and Computer Science, University of the Balearic Islands, Palma de Mallorca, Spain; University of Vermont, UNITED STATES

## Abstract

*Reactive navigation* is a well-known paradigm for controlling an autonomous mobile robot, which suggests making all control decisions through some light processing of the current/recent sensor data. Among the many advantages of this paradigm are: 1) the possibility to apply it to robots with limited and low-priced hardware resources, and 2) the fact of being able to safely navigate a robot in completely unknown environments containing unpredictable moving obstacles. As a major disadvantage, nevertheless, the reactive paradigm may occasionally cause robots to get trapped in certain areas of the environment—typically, these conflicting areas have a large concave shape and/or are full of closely-spaced obstacles. In this last respect, an enormous effort has been devoted to overcome such a serious drawback during the last two decades. As a result of this effort, a substantial number of new approaches for reactive navigation have been put forward. Some of these approaches have clearly improved the way how a reactively-controlled robot can move among densely cluttered obstacles; some other approaches have essentially focused on increasing the variety of obstacle shapes and sizes that could be successfully circumnavigated; etc. In this paper, as a starting point, we choose the best existing reactive approach to move in densely cluttered environments, and we also choose the existing reactive approach with the greatest ability to circumvent large intricate-shaped obstacles. Then, we combine these two approaches in a way that makes the most of them. From the experimental point of view, we use both simulated and real scenarios of challenging complexity for testing purposes. In such scenarios, we demonstrate that the combined approach herein proposed clearly outperforms the two individual approaches on which it is built.

## Introduction

To introduce this work, in this section, we overview robot control paradigms, distinguish among the different kinds of reactive control approaches, consider relevant contributions in this field, and, finally, set the goals of this research.

### Brief overview of robot control paradigms

One of the fundamental challenges posed to mobile robotics is the task of navigating autonomously and safely from one place to another in the presence of obstacles. To face this challenge, roboticists have tried to emulate in robots the behaviors and responses to stimuli demonstrated by living creatures. As a result of this effort, three primary paradigms for robot control have essentially emerged, named *deliberative*, *reactive*, and *hybrid*. Roughly speaking, these paradigms differ from each other in how sensory data is processed and distributed through the control system, and also where decisions are made. In other words, each of these paradigms describes a different relationship among the primitive functions Sense, Plan, and Act. In the following, we detail further the essentials, as well as the strengths and weaknesses, of the deliberative, reactive, and hybrid robot control paradigms (see [1] in case the reader wants to learn still more about these paradigms):

- As for **the deliberative paradigm**, it mainly focuses on heavy reasoning and knowledge representation. To be more precise, a deliberatively-controlled robot operates in a top-down fashion by repeating the following sense-plan-act cycle: first of all, the robot senses the world and integrates the sensed data into a global world model; afterwards, based on such a model, the robot computes/plans the series of actions needed to get to the desired destination from its current position; and, at last, the planned actions are dispatched for execution. The deliberative paradigm has important limitations. More specifically, it just works well when the so-called closed-world assumption is met. This assumption requires that there are no unpredictable situations, and also that large amounts of computing power are available. Unfortunately, in practice, these requirements are too severe since, on the one hand, the majority of the real-world environments are inherently dynamic and, on the other hand, the use of low-cost robots—with low-performance computational units— is becoming mandatory to allow robotics to enter the domestic and service markets.
  *Shakey* [2] was one of the first general-purpose mobile robots capable of reasoning about its own actions. This was achieved by controlling Shakey according to the deliberative paradigm.
- As for **the reactive paradigm**, it advocates for a direct connection between sensing and action, mediated neither by heavy reasoning nor by knowledge representation (that is why the reactive paradigm is also known as the sense-act paradigm). Or in other words, under this paradigm, the real world is considered to be its own best model; in consequence, reactively-controlled robots do not operate upon abstract representation of reality, but upon the real world itself. As a major advantage, the reactive paradigm removes the need for the aforementioned closed-world assumption. Through experimentation, this paradigm has demonstrated to provide quick reaction times in unpredictable situations—typically caused by unknown and moving obstacles—, even when being run on low-cost robots. However, the reactive paradigm has disadvantages as well, all of them derived from the fact that decisions about the robot’s actions are now made using local, rather than global—as in the deliberative paradigm—, information of the environment. In short, these disadvantages are: (1) a reactively-controlled robot has difficulties to navigate in complex environments (by way of example, these robots get frequently trapped in concave obstacle configurations such as the typical U-shaped canyon); and (2) the path followed by a reactively-controlled robot during the navigation is usually highly suboptimal, in terms of length and/or clearance and/or smoothness and/or any other quality criterion.
  *Rodney Brooks* is recognized as a forefather of the reactive paradigm. In 1986, he presented an inspiring paper entitled “Intelligence Without Representation” [3] that questioned the necessity of using explicit representations and models of the world for building intelligent autonomous robots. As a direct consequence of this, a few years later, Brooks proposed one of the most well-known reactive control architectures [4]. This architecture, which was given the name of *Subsumption*, provided robots with a set of simple stimulus-response reflexes. From the interaction among these reflexes, robots could perform behaviors similar to those observed in insects.
- Finally, the essence of **the hybrid paradigm** is to combine the reasoning capabilities of the deliberative paradigm with the responsive capabilities of the reactive paradigm. While the deliberative paradigm relies on the idea of sense-plan-act and the reactive paradigm follows a sense-act philosophy, the hybrid paradigm takes the form of plan, sense-act, with the comma meaning parallel execution. In its basic conception, a control system based on the hybrid paradigm makes use of two decoupled layers of functionality, one deliberative and the other reactive. The deliberative layer provides a global plan as input to the reactive layer. Then, the reactive layer executes this global plan within its local context and according to its local constraints. Besides, it is important to note that, during the execution of the global plan, the deliberative layer is allowed to interrupt the reactive layer if better plans are discovered. Or what is more, in order to ensure responsiveness, partial plans may also be given to the reactive layer when there is no time to wait for the complete solution. As a key advantage, the hybrid paradigm enables robots to perform highly complex navigation tasks, such as those found in the area of field robotics where robots are required to move through the real outdoor world—i.e. through environments that are unstructured, partially or totally unknown, constantly changing, and full of uncertainty (as a successful example of the application of the hybrid paradigm for the resolution of a complex navigation task, the winner of the DARPA Urban Challenge, a robot called *Boss* [5], was able to autonomously navigate over 60 miles of urban terrain by using a layered control system that contained both deliberative and reactive components). Regarding the disadvantages of the hybrid paradigm, one of the most problematic issues is the difficulty that exists to achieve the right compromise between the deliberative and reactive layers. The compromise between global objectives and local constraints is not always easy to find, and often the tradeoffs have to be empirically “fine-tuned” by the robot software designer. This process of fine-tuning may be time-consuming, or what is even worse than that, it may be very dependent on the target environment—if so, such a fine-tuning process should be repeated each time the target environment changes. As a last note, another problematic issue of the hybrid paradigm comes from the fact of having included a layer for global/high-level deliberation. In practical terms, deliberation means the need for substantial hardware resources, especially large amounts of memory and significant computational power. Unfortunately, this necessity is not supported by the majority of low-cost robots, which are equipped with very limited hardware resources, such as microcontrollers.
  *James Albus* and *Anthony Barbera* were two of the first roboticists in designing a control system—known as *Real-time Control System*, or *RCS* for short— which mixed both reactive and deliberative components [6].

Control systems operating under the deliberative, reactive, and hybrid paradigms can all be effective depending on the context in which they are used. In this respect, nevertheless, it is fair to point out that most modern control systems are either reactive or hybrid (the deliberative paradigm has practically fallen into disuse, probably because its underlying assumptions conflict with reality). Moreover, due to the difficult design and tuning of hybrid control systems as well as to the always-present goal of trying to find the perfect balance between effort and results, nowadays the hybrid paradigm is strictly used just when the reactive paradigm is not good enough for a satisfactory robot performance. As a general idea about the specific domains where the reactive and hybrid paradigms are currently being applied by researches in robotics, it can be said that: when the intended navigation task is “simple”, there is a marked preference for the use of the reactive paradigm; however, when the task is “complex”, the use of the hybrid paradigm becomes mandatory since the reactive paradigm does not offer a solution to the problem. Table 1 summarizes the major advantages and disadvantages of the deliberative, reactive, and hybrid paradigms. Furthermore, Table 2 explains in which cases it is preferable to use one paradigm over the others.

**Table 1:** Main advantages and disadvantages of the three prevailing paradigms for robot control.

Deliberative Paradigm	Reactive Paradigm	Hybrid Paradigm
**Advantages**		
Navigation tasks of high complexity can be efficiently performed, but just in static environments Robots are provided with a high level of skill and intelligence	Use of a partial and simplified model of the world (note that, in the *pure* version of this paradigm, there is a total absence of a world model) Use of limited computational power and memory Suitable for low-cost robots The closed-world assumption is not necessary, because the robot is able to quickly react to unpredictable situations	Navigation tasks of high complexity can be efficiently performed, even in dynamic environments Robots are provided with a high level of skill, intelligence, and responsiveness
**Disadvantages**		
Large amounts of memory are required to maintain a very detailed and accurate model of the world A high computational power is required to make decisions based on the world model Not suitable for low-cost robots, i.e. for robots with limited resources Safe navigation is only guaranteed in environments where the—unrealistic— closed-world assumption is met	Decisions are made with no global knowledge, which may lead to situations where the robot gets to the desired target through a *poor* path, or, to make matters worse, may cause the robot to fail in reaching the target (here, the word “poor” essentially means: a path that is much longer and far less smooth than the best possible path)	Complex design: the control of the robot is achieved through a complex internal structure composed of several functional layers—the deliberative layer, the reactive layer, and some other layers—that interact with one another Large amounts of memory are required to maintain the global world model used by the deliberative layer, and the local world model used by the reactive layer A high computational power is required to allow the deliberative and reactive layers to make decisions based on their world models Not suitable for low-cost robots

**Table 2:** A set of rules to decide when to apply a particular paradigm.

Deliberative Paradigm	Reactive Paradigm	Hybrid Paradigm
When to use it		
When a robot equipped with high processing and memory capabilities is available When the navigation task to be performed is complex When it is important that the robot efficiently performs the navigation task When the robot is going to navigate only in static environments	When a robot equipped with low processing and memory capabilities is available When the navigation task to be performed is simple When no matter how efficiently the robot performs the navigation task When the robot is going to navigate in both static and dynamic environments	When a robot equipped with high processing and memory capabilities is available When the navigation task to be performed is complex When it is important that the robot efficiently performs the navigation task When the robot is going to navigate in both static and dynamic environments

In the light of the above discussion, one can easily imagine the benefits that will come from extending the currently-restricted domain of application of the reactive paradigm. Following this, it seems clear that if, for solving non-simple navigation tasks, there was not the absolute necessity of applying the hybrid paradigm, or equivalently, it was possible the application of the reactive paradigm, far less time and effort—and, therefore, costs—should be spent designing, developing, and testing the robot control system.

With this motivation in mind, in this paper, we propose a new method for reactive autonomous robot navigation whose principal objective is that of performing more complex tasks than what has been exhibited up-to-now by state-of-the-art methods. Next, we will go deeper into the reactive paradigm. To be more precise, we first look at the most widely-known classification of reactive control systems. Afterwards, we describe the best currently-existing methods within each of the classification categories.

### General classification of reactive control systems

#### Pure versus non-pure

As we have already mentioned before, a reactive control system is characterized by a tight coupling of sensing to action, typically involving a very small amount of computation/reasoning, and very simple—if any—internal representations of the world where navigation is to occur. In the robotics literature, the fact of having, or not having, internal world representations has been used as a criterion to distinguish different types of reactive control systems. According to this criterion, there exist two types of reactive control systems, namely *pure* and *non-pure*. In essence, a reactive control system is said to be “pure” when no use is made of internal world representations; otherwise, the reactive control system is considered as “non-pure”. Let us now discuss these concepts in a little more detail:

- **Purely reactive control systems** are systems without memory, which means that they react directly to current sensor data. That is to say, in these systems, the actions performed at time *t*_*i*_ are decided on the basis of, solely, the sensor readings taken at time *t*_*i*_.
- **Non-purely reactive control systems** are systems that provide robots with a short-term memory of the most recent sensor data. This short-term memory can be seen as an internal representation of the world in the close surroundings of the robot (although obvious, the larger the memory, the larger the view of the robot’s surroundings). In these systems, actions are completely determined by the information contained in the short-term memory. Or said in other words, assuming the use of a short-term memory of size *N*, the actions taken at time *t*_*i*_ are decided on the basis of the sensor readings taken from time *t*_*i*−*N*+1_ to *t*_*i*_.

As a final point, it should be noted that non-purely reactive control systems usually perform much better than their purely reactive counterparts, but at the cost of requiring some additional memory and computation time.

In the following, we discuss how the value of *N* influences on the performance of a non-purely reactive control system.

#### The choice of *N* as a trade-off between effectiveness and reactiveness in non-purely reactive control systems

Generally speaking, the iteration cycle of a non-purely reactive control system consists of three main steps: first of all, data from the robot’s sensors are gathered and temporally accumulated in a short-term memory (regarding the meaning that the word “temporally” has here, it is important to note that each data will remain stored in the short-term memory for just *N* iteration cycles, being *N* ≥ 1); as a second step, based on both the current situation of the robot—this is supposed to be known/estimated from the information available in the short-term memory—and the set of goals to be reached by the robot during navigation, it is computed the best action that should happen next; and, finally, the previously computed action is sent to the robot’s actuators for execution. These steps are repeated over and over at a high frequency until all navigation goals are successfully fulfilled.

With the above in mind, it is worth mentioning that the value given to *N* has a strong influence on the performance that non-purely reactive control systems exhibit when carrying out navigation tasks. In short, the more the value of *N*, the richer the information on which decisions are made, and, therefore, the higher the probability that these local decisions translate into effective global actions. Merely according to this, it seems that the value of *N* should be chosen as high as possible. However, the truth is that one has to choose a compromise value for *N*. This is due to the fact that the more the value of *N*, the more the memory which is required to hold sensor data—because the short-term memory retains all sensor data collected over the last *N* iteration cycles—, and the more the expense of computation time for making decisions—because these decisions would be made on the basis of a larger amount of information—(note additionally that an increase of computation time does imply a loss of reactiveness). From all that has been said, we can briefly conclude that, in non-purely reactive control systems, the value of *N* allows establishing the desired trade-off between, essentially, the two following competing interests: effectiveness and reactiveness.

In most cases, non-purely reactive control systems treat *N* as an external parameter which has to be set, by a user—with some expertise—, to any desired value before navigation begins. On the other hand, although less common, there are also non-purely reactive control systems that consider *N* as an internal parameter. In such a case, it is important to understand that the system assumes the entire responsibility for giving a proper value to *N* prior to starting the navigation task. Moreover, in their great majority, these systems manage *N* adaptively; i.e. the value they initially give to *N* does not remain constant over time, but rather it is continually adjusted to adapt to current circumstances of navigation. As an outstanding example of that adjustment, note that when a system with internal and adaptive parametrization of *N* detects the robot may be currently trapped by an obstacle, the value of *N* is immediately increased with the aim of improving the understanding the system has about the robot’s surroundings, or what is more, in order that this better understanding helps the system to find—if any—new paths which allow the robot to escape from/circumnavigate the blocking obstacle (as a final remark on this example, let us mention that *N* is usually upper bounded, which means its value cannot be increased beyond a certain maximum; by doing so, a limit is actually imposed on the amount of reactiveness a system of this type is permitted to sacrifice in favor of a more effective behavior of the robot; as one may well imagine, such a limit should be chosen carefully, in a way that ensures the robot can sufficiently react to the most likely unexpected events). As a counterpoint to the previous example, note additionally that when a system with internal and adaptive parametrization of *N* detects the robot is currently surrounded by small-sized, simple-shaped and sparse-distributed obstacles, the value of *N* is immediately decreased, because it is assumed that a basic understanding of the robot’s surroundings—with the word “basic” meaning here that *N* takes a low value equal to or slightly higher than 1—will be enough so that the system can make the robot successfully navigate around all such—minor—obstacles; or said differently, in a context where obstacle avoidance can be effectively accomplished with only a reduced amount of local information about the environment, the system aims to improve reactiveness, thus making the robot more capable of handling the unexpected.

### Relevant contributions in the development of reactive robots

Here, we briefly describe some of the most outstanding approaches to autonomously control a mobile robot conforming to the reactive paradigm. In order to facilitate this description, we have grouped the approaches into different classes (A, B, …) based on their operating principles.

Class A The first class comprises those approaches that construct an **artificial potential field**. This artificial potential field is defined as the sum of attractive forces, pulling the robot towards the desired target location, and repulsive forces, pushing the robot away from obstacles. The resultant force is expected to drive the robot to the target through a safe path. *APF* (*Artificial Potential Field*) [7] and *VFH^⋆^* (star version of the *Vector Field Histogram*) [8] are two representative approaches of this class.
  The approaches of A-class have proven to be a very simple, fast, and effective way of implementing reactive control over a robot. These approaches, nevertheless, are known to suffer from important drawbacks [9]. Among these drawbacks, we emphasize the following: (1) oscillations occur when the robot travels in narrow passages (this is a serious drawback that may cause the robot to collide with obstacles); (2) the sum of the attractive and repulsive forces may be zero in a point other than the target (this means that the robot may get trapped in local minima).
  Recently, there have been some attempts to solve the above-mentioned drawbacks of artificial potential fields, as evidenced by the works [10] and [11].
Class B The second class comprises those approaches that rely on the so-called **curvature velocity assumption**. Under this assumption, a robot is supposed to move exclusively along circular arcs and straight lines. In each control cycle, a motion command is computed by searching the point in the velocity space (*v*, *w*) that maximizes an objective function which trades-off speed, safety, and target-directness. *CVM* (*Curvature-Velocity* Method) [12], *DWA* (*Dynamic Window* Approach) [13], *BCM* (*Beam Curvature* Method) [14], *PBCM* (*Prediction-based Beam Curvature* Method) [15], and *DCVM* (*Dynamic Curvature-Velocity* Method) [16] are some approaches belonging to this class.
  The B-class approaches share common advantages and disadvantages. As for the advantages, these approaches allow robots to navigate at a high speed with no risk of collision, since they take into account the kinematic and dynamic constraints of the robot (more specifically, they make velocities and accelerations bounded and compatible with those that the robot can perform). On the other hand, among the disadvantages of these approaches, we find the following: (1) their limited scope of application, which is essentially restricted to synchro-drive and differential-drive robots; and (2) the difficulty in obtaining an appropriate trade-off among the three conflicting terms—speed, safety, and target-directness—included in the objective function (this should be done by assigning a weight to each term, but the choice of values is purely empirically based).
Class C The third class comprises those approaches that are founded upon **the notion of opening or gap**. Shortly speaking, a gap is defined as a free space between two obstacles large enough for the robot to cross through. In essence, these approaches operate in two basic steps: first, they search for all gaps by analyzing the discontinuities in the robot’s current field of view; later, they choose the gap which is more likely to drive the robot towards the desired target location. *ND* (*Nearness Diagram*) [17] and *CG* (*Closest-Gap*) [18] are two representative approaches of this class.
  The approaches of C-class are known to be capable of safely moving a robot among very closely-spaced obstacles. Unfortunately, these approaches also have disadvantages, being the most important one the fact of not guaranteeing the reachability of the target in environments having obstacles bigger in size than the robot’s local field of view.
Class D The fourth class comprises those approaches that belong to the so-called **family of Bug algorithms**. All algorithms in this family generically operate as follows: as a first step, the robot is moved directly towards the desired target location; this is done until finding an obstacle that prevents the robot from progressing; at that moment, a second step starts, which consists of making the robot follow the boundary of the blocking obstacle until a certain condition is met—this condition is typically referred to as the *leaving* condition; when the leaving condition is satisfied, the first step is executed again. This two-step iterative process is repeated until the robot reaches the target.
  Bug algorithms fundamentally differ from each other in the way they define the leaving condition. *Bug1* was the first Bug algorithm, and was proposed by professors Lumelsky and Stepanov in 1986 [19]. Since then, the number of Bug algorithms has never stopped growing. Some widely-known members of the Bug family are [20–24].
  The main advantage of Bug algorithms is that they are able to guarantee *completeness*; that is to say, they ensure the robot will get to the target if a path exists. Regarding the disadvantages of Bug algorithms, the following have been reported: (1) the environment is assumed to contain just static obstacles; (2) the robot is assumed to be a point (this actually means that the robot has no size and can fit through any arbitrarily small gap between two obstacles); (3) the robot is assumed to have perfect localization capabilities as well as very precise sensors for detecting obstacles. Obviously, these three assumptions are quite unrealistic.
Class E The fifth and last class comprises those **approaches designed to be integrated into others**. Broadly speaking, an approach of E-class can be thought of as a component which carries with it specific abilities from the viewpoint of robot navigation. When one of these components is added to an existing reactive approach, this approach expands its abilities with those inherent to the component.
  T^2^ is an example of E-class component [25]. More specifically, this component gives reactive approaches the ability to move a robot through environments with complex obstacles without getting stuck. As may be noted in [26] and [27], T^2^ has been successfully integrated into approaches of A- and B-class with the aim of avoiding both local trap situations and cyclic behaviors.

The approaches of A-, B- and C-class are purely reactive, whereas the approaches of D- and E-class are non-purely reactive.

### Research objectives and structure of the paper

In this paper, as a starting point, we choose the existing reactive approach with the greatest ability to move in densely cluttered environments—i.e. the best approach of C-class; we also choose the existing reactive approach with the greatest ability to avoid local trapping situations and cyclic behaviors—in this case, the best approaches are found in classes D and E. Then, we combine these two approaches to make the most of them.

The results presented in [28] leave no doubt about what is the best approach of C-class. Currently, the approach named *Tangential Gap Flow* (TGF) outperforms any other approach within the scope of “motion in cluttered environments”. On the other hand, the comparative study conducted in [25] together with the task of literature review that the authors of this paper have carried out of the last five years of research allow us to affirm that T^2^ is one of the best approaches to significantly increase the probability that the robot gets to the target, always under the rules dictated by the reactive paradigm and without making any unrealistic assumption.

The rest of the paper is organized as follows: firstly, we will discuss about how the TGF and T^2^ strategies operate; secondly, we will propose a way to merge the two aforementioned strategies; thirdly, we will test the strategy resulting from the merging in both simulated and real scenarios; fourthly, we will discuss the experimental results; and, finally, we will end the paper by drawing some conclusions and possible future research lines.

## Overview of the operation of TGF and T^2^

### The Tangential Gap Flow (TGF) strategy

*Closest-Gap* is the generic name of a family of reactive control strategies of pure type which are intended to make a robot move safely in narrow spaces, i.e. in places where the distance between obstacles is barely bigger than the physical size of the robot.

This family takes its name from its first strategy, which was published in 2006 [18]. This strategy, briefly called *CG*, was proposed as an improvement of the *Nearness Diagram* (*ND*) [17] and the *Smooth Nearness Diagram* (*SND*) [29] methods. CG, just like ND and SND, is capable of navigating a robot through closely-spaced obstacles. Nevertheless, CG is a step ahead of ND and SND in various aspects: first of all, from a computational point of view, CG is less demanding—decisions are made by involving a slightly less complex reasoning; secondly, CG produces smoother robot movements; and, finally, CG decreases the probability that the robot is trapped in a position other than the target.

Very soon after the publication of CG, the Closest-Gap family continued to grow, and two new strategies were put forward, named *Safe Gap* (*SG*) [30] and *Tangential Closest Gap* (*TCG*) [31]. SG enhances the safety of the robot trajectories produced by CG. To this end, SG deeply analyses how obstacles are distributed between the robot and the navigable opening which is closest to the robot. After this analysis, SG drives the robot through such a navigable opening by generating a sequence of subgoals, whose path to them is ensured to be free of obstacles. On the other hand, TCG produces faster, shorter and less oscillatory robot trajectories than CG. To achieve all these improvements, TCG essentially makes the robot navigate parallel to the tangent of the closest obstacle, while progressing towards the target. What is more, TCG sends motion commands to the robot which are proven to be stable in Lyapunov terms.

The last strategy in joining the Closest-Gap family has been *Tangential Gap Flow* (*TGF*) [28]. This strategy tries to combine the high robot safety level provided by SG with the more efficient and more stable robot behavior exhibited by TCG. The following sections describe how the TGF strategy operates, and also include a discussion about what the strengths and weaknesses of TGF are.

#### Some operating details

The operation of TGF can be thought of as being divided into three sequential stages. In the first stage—*S*_1_—, TGF searches for collision-free paths (although obvious, note that this search is *local* in nature, which means it is performed by using, exclusively, the information about the environment that the robot’s sensors provide at the moment in which the search is started). In the second stage—*S*_2_—, TGF chooses one collision-free path from all of those found in the previous stage. At last, in the third stage—*S*_3_—, TGF generates the motion commands that make the robot follow the path which was chosen in stage *S*_2_. The execution of stages *S*_1_ to *S*_3_ is repeated until the robot completes its task, i.e. until the robot reaches the desired target position. A more in-depth view of how TGF operates is provided next.

**Fig 1:**
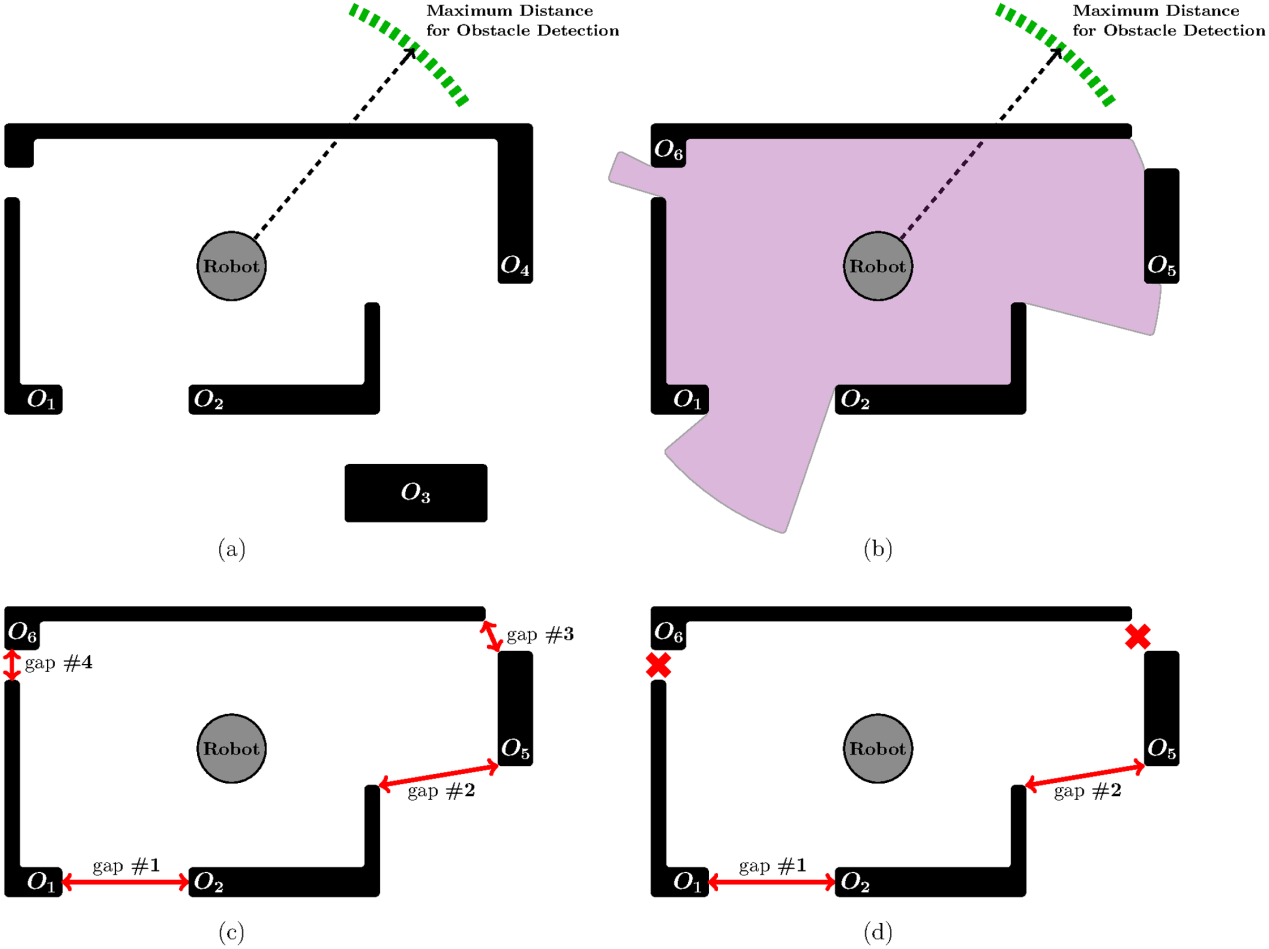
TGF in action: An example showing the execution of stage *S*_1_. (a) the environment where the robot should navigate (obstacles are represented by the thick black lines as well as the black rectangle; the robot is assumed to be equipped with a laser scanner which measures the distance to all surrounding obstacles in a 360-degree field of view); (b) the environment as locally perceived by the robot; (c) detection of gaps between obstacles; (d) gaps #3 and #4 are eliminated, because these gaps are narrower than the robot.

In stage *S*_1_, TGF analyses the immediate surroundings of the robot in search of the so-called *gaps* (as explained in [28], a *gap* is a space between two obstacles wide enough for the robot to navigate through). Strictly speaking, stage *S*_1_ is made of the following steps: first—*S*_1.1_—, TGF collects data from the environment by using the sensors that are mounted on the robot; thereafter—*S*_1.2_—, TGF makes use of the information obtained in the previous step to find gaps (at this point, it is important to highlight that any gap is considered, regardless of its size); finally—*S*_1.3_—, the set of gaps found in step *S*_1.2_ are filtered by removing the gaps narrower than the physical size of the robot. Fig 1 illustrates an example of the three steps performed in stage *S*_1_.

Stage *S*_2_ receives as input the set of gaps surviving step *S*_1.3_. Here, from all these gaps, TGF chooses the one that is likelier to allow the robot to reach—or, at least, get a bit closer to—the desired target position. To discuss this idea in more detail, we first need to introduce a few concepts: let *C* denote the center of the robot platform, *T* the target point, *l*_*T*_ the straight-line segment that connects *C* to *T*, $p_{1}^{i}$ and $p_{2}^{i}$ the endpoints of gap #*i*, $l_{j}^{i}$ the straight-line segment that joins *C* to $p_{j}^{i}$ for any *j* ∈ {1, 2}, and, lastly, *dist*(*l*_*A*_, *l*_*B*_) is a function that returns the angle between the straight-line segments *l*_*A*_ and *l*_*B*_, and *mindist*^*i*^ is another function defined by:

$$\begin{array}{c} min(dist(l_{T},l_{1}^{i}),dist(l_{T},l_{2}^{i})). \end{array}$$ (1)

According to expression 1, given gap #*i*, the *mindist*^*i*^ function finds the minimum angular distance between *l*_*T*_ and each of the straight-line segments $l_{1}^{i}$ and $l_{2}^{i}$.

**Fig 2:**
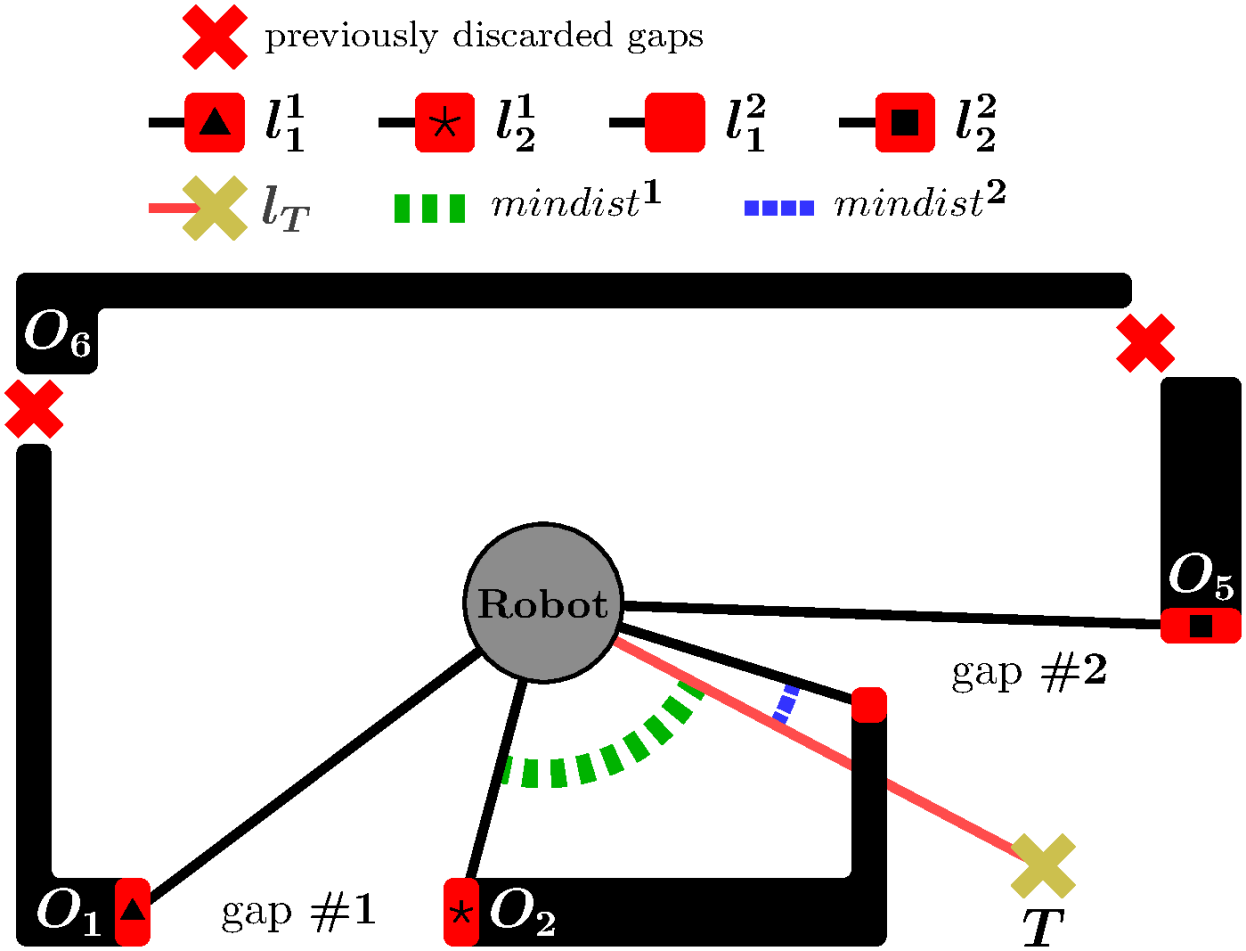
TGF in action: An example showing the execution of stage *S*_2_. Gap #2 is selected instead of gap #1 because *mindist*^2^ < *mindist*^1^.

After introducing these concepts, we can go back to the explanation of stage *S*_2_. In this stage, TGF does choose the gap with the smallest *mindist*^*i*^. Fig 2 shows graphically how the above-described process of gap selection really works (as a continuation of the example of Fig 1).

Turning our attention to stage *S*_3_, TGF sends a control command to the robot as a 2-tuple: (steering direction, speed). Roughly speaking, such a motion command tries to guide the robot along a collision-free path towards the gap selected in stage *S*_2_. What is more, while following this path, the robot is constrained to be kept in the midst of the free space left by those obstacles located at both sides, and it is also constrained to move parallel to the tangent of the obstacle which is closest to the robot. (Further details about how TGF computes this control command can be found in [28]).

#### Strengths and weaknesses

In the light of the results presented in [28], where TGF is compared against the ND, SND and CG strategies in dense and cluttered environments, we can justifiably claim that TGF presently outperforms all competitors because it is able to generate faster, shorter and smoother robot trajectories.

**Fig 3:**
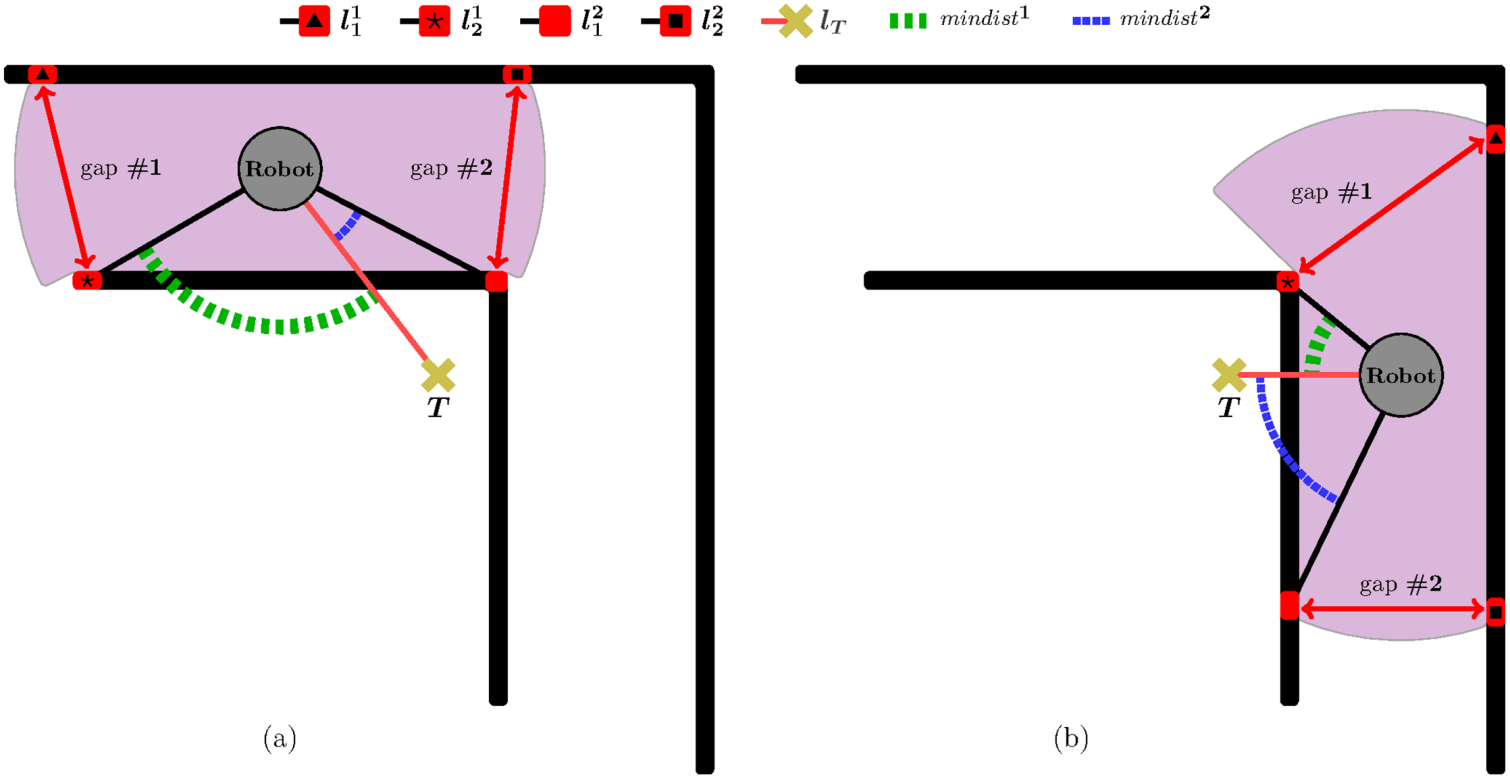
A scenario where TGF fails to guide the robot to the target.

Unfortunately, TGF has a major drawback, which is shared by all purely reactive approaches. A robot controlled by TGF may become trapped in front of an obstacle or may wander indefinitely in a confined region of the environment, being unable to get to the given target point. By way of example, let us assume that the robot is in the environment shown in Fig 3(a), at the position indicated by the big gray circle. In such a situation, the TGF strategy will steer the robot towards the gap #2; or, in other words, TGF will decide to try to reach the target by following the corridor in a clockwise direction. This decision will be effective until the robot is at the position of Fig 3(b). At that moment, the TGF strategy will steer the robot towards the gap #1; or, in other words, TGF will decide to try to reach the target by following the corridor in a counterclockwise direction. This decision will change again when the robot arrives at the position of Fig 3(a), and, consequently, the robot gets into a cyclic path.

### The T^2^ strategy

In 2005, the authors of the present work published a paper reporting a novel control strategy called *T^2^* [26]. The aim of this strategy was enabling robots to rapidly make intelligent decisions by exploiting the power offered by the non-pure reactive paradigm. The term *T^2^ = TT* comes from the two basic principles which govern the operation of the strategy. More specifically, these principles are: the principle of ***T****raversability* and the principle of ***T****enacity*. By applying the principles of Traversability and Tenacity, a robot does acquire the ability to avoid getting stuck against large obstacles, even when these obstacles are intricate (in this respect, let us note that: (*i*) a *large* obstacle goes beyond the robot’s neighbourhood; or, put differently, a large obstacle is one that can only be partially perceived through the robot’s sensors, because the obstacle is so big in size that some parts of it are not within the detection range—this fact constitutes a serious disadvantage since it makes difficult for the robot to take decisions that help in overcoming such large obstacles; (*ii*) an *intricate* obstacle involves the existence of a region in the environment that is easy to get into, but very difficult to get out of; (*iii*) an obstacle which is both, large and intricate, suffers from all the disadvantages indicated above). The last version of the T^2^ strategy was published in [25] after years of improvements. This section describes the T^2^ strategy as it appears in [25].

As has been previously mentioned, the T^2^ strategy is conceived under the principles of Traversability and Tenacity. Hence, we will first introduce the Traversability and Tenacity principles, and, later, we will discuss about the behaviour that a robot exhibits when it is controlled in accordance with these two principles. Lastly, we will address the strengths and weaknesses of the T^2^ strategy.

#### The Principle of Traversability

**Fig 4:**
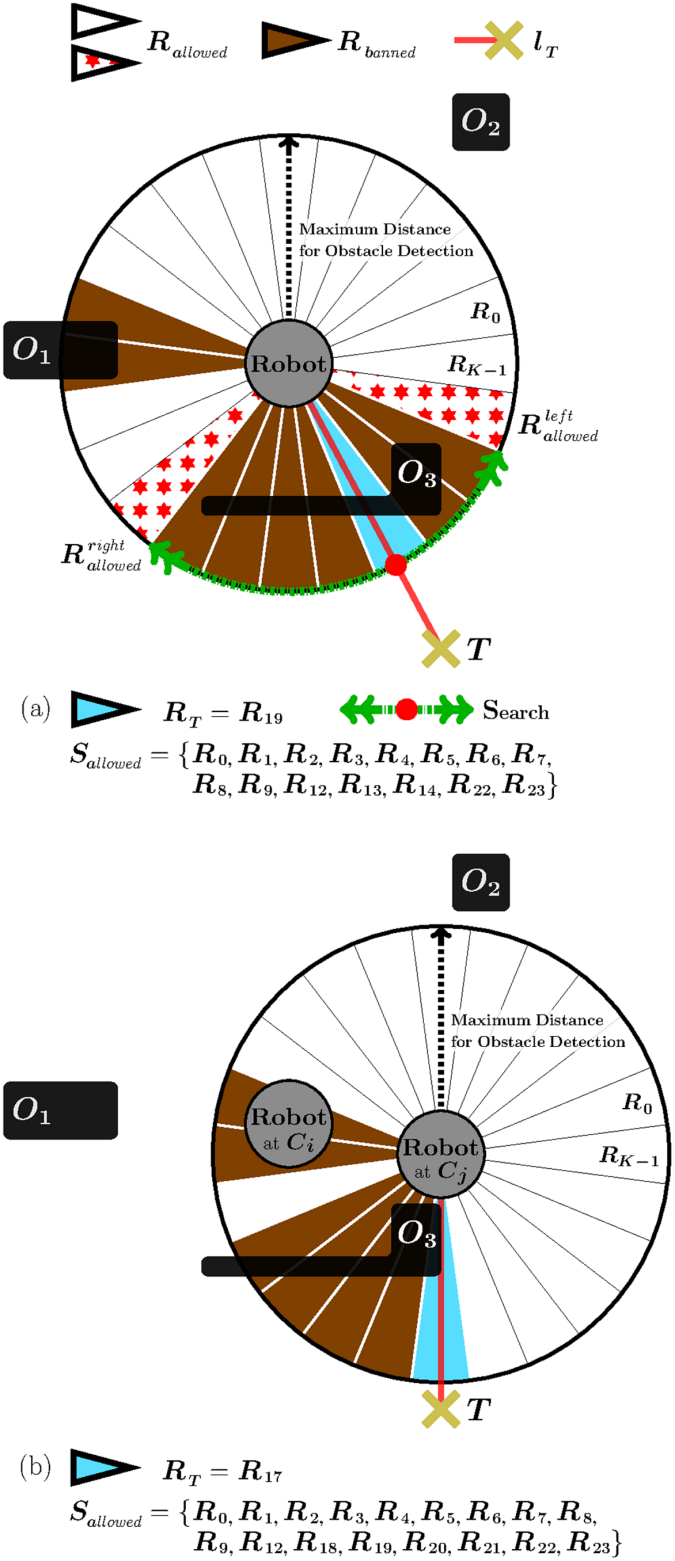
Exemplification of the *Traversability* principle assuming *K* = 24.

The principle of Traversability materializes through the four following features (features 1 to 3 are illustrated in Fig 4(a), while feature 4 is shown in Fig 4(b)):

Feature 1 The local environment of the robot is divided into *K* angular regions, *R*_0_ … *R*_*K*−1_, of equal size.
Feature 2 Each angular region is labelled as either *allowed* or *banned*. Given an *i* ∈ {0, …, *K* − 1}, the angular region *R*_*i*_ is said to be allowed when the robot does not detect the presence of any obstacle within *R*_*i*_ based on the current sensor readings. Otherwise, *R*_*i*_ is said to be banned.
Feature 3 Let *R*_*T*_ be the angular region in which *l*_*T*_ falls (*l*_*T*_ is the straight-line segment between the robot’s position—*C*—and the target point—*T*). Additionally, let *S*_*allowed*_ be the set of all angular regions labelled as allowed. Then, $R_{allowed}^{right}$ and $R_{allowed}^{left}$ are defined as follows:
  $$\begin{array}{c} R_{allowed}^{right}=\{\begin{array}{cc} \text{if}\;R_{T}\in S_{allowed}, & R_{allowed}^{right}=R_{T}\\ \text{otherwise}, & R_{allowed}^{right}\;\text{is}\;\text{the}\;\text{angular}\;\text{region}\;\text{of}\;S_{allowed}\\ & \text{that}\;\text{is}\;\text{closest}\;\text{to}\;R_{T}\;\text{clockwise.} \end{array} \end{array}$$ (2)
  $$\begin{array}{c} R_{allowed}^{left}=\{\begin{array}{cc} \text{if}\;R_{T}\in S_{allowed}, & R_{allowed}^{left}=R_{T}\\ \text{otherwise}, & R_{allowed}^{left}\;\text{is}\;\text{the}\;\text{angular}\;\text{region}\;\text{of}\;S_{allowed}\\ & \text{that}\;\text{is}\;\text{closest}\;\text{to}\;R_{T}\;\text{counterclockwise.} \end{array} \end{array}$$ (3)
Feature 4 A short-term memory reminds the robot of the obstacles that were detected recently. Fig 4(b) depicts this idea assuming that the robot has moved from *C*_*i*_ to *C*_*j*_ (note that *C*_*i*_ is the position where the robot was in Fig 4(a)). Looking at Fig 4(b), the following observations can be made: (*i*) the obstacle *O*_1_ is sensed by the robot at *C*_*i*_; (*ii*) when the robot is located at *C*_*j*_: (*ii*.*a*) the obstacle *O*_1_ is not within the robot’s sensing range; (*ii*.*b*) in spite of observation (*ii*.*a*), the use of a short-term memory allows the robot to remember the presence of the obstacle *O*_1_—this is evidenced by the fact that regions *R*_10_ and *R*_11_ are banned.

By way of summary, we can say that, as a result of applying the principle of Traversability, the T^2^ strategy selects two regions: $R_{allowed}^{right}$ and $R_{allowed}^{left}$.

#### The principle of Tenacity

The second principle, known as Tenacity, aims at selecting one of the two regions produced by the principle of Traversability: $R_{allowed}^{right}$ or $R_{allowed}^{left}$.

The principle of Tenacity is defined in two different ways, which go under the names of *right-hand tenacity* and *left-hand tenacity*. These definitions differ from one another in the criterion used to choose between $R_{allowed}^{right}$ and $R_{allowed}^{left}$. Simply expressed, right-hand tenacity always chooses $R_{allowed}^{right}$, while left-hand tenacity always selects $R_{allowed}^{left}$. A user-definable parameter determines the type of tenacity, either right-hand or left-hand, we want to apply.

Henceforth, we will generically denote by *R*_*ten*_ the region which has been selected according to the principle of Tenacity, and by *l*_*ten*_ the straight line with origin at *C* that divides *R*_*ten*_ into two equal parts.

#### The behaviour that naturally emerges from the principles of Traversability and Tenacity

**Fig 5:**
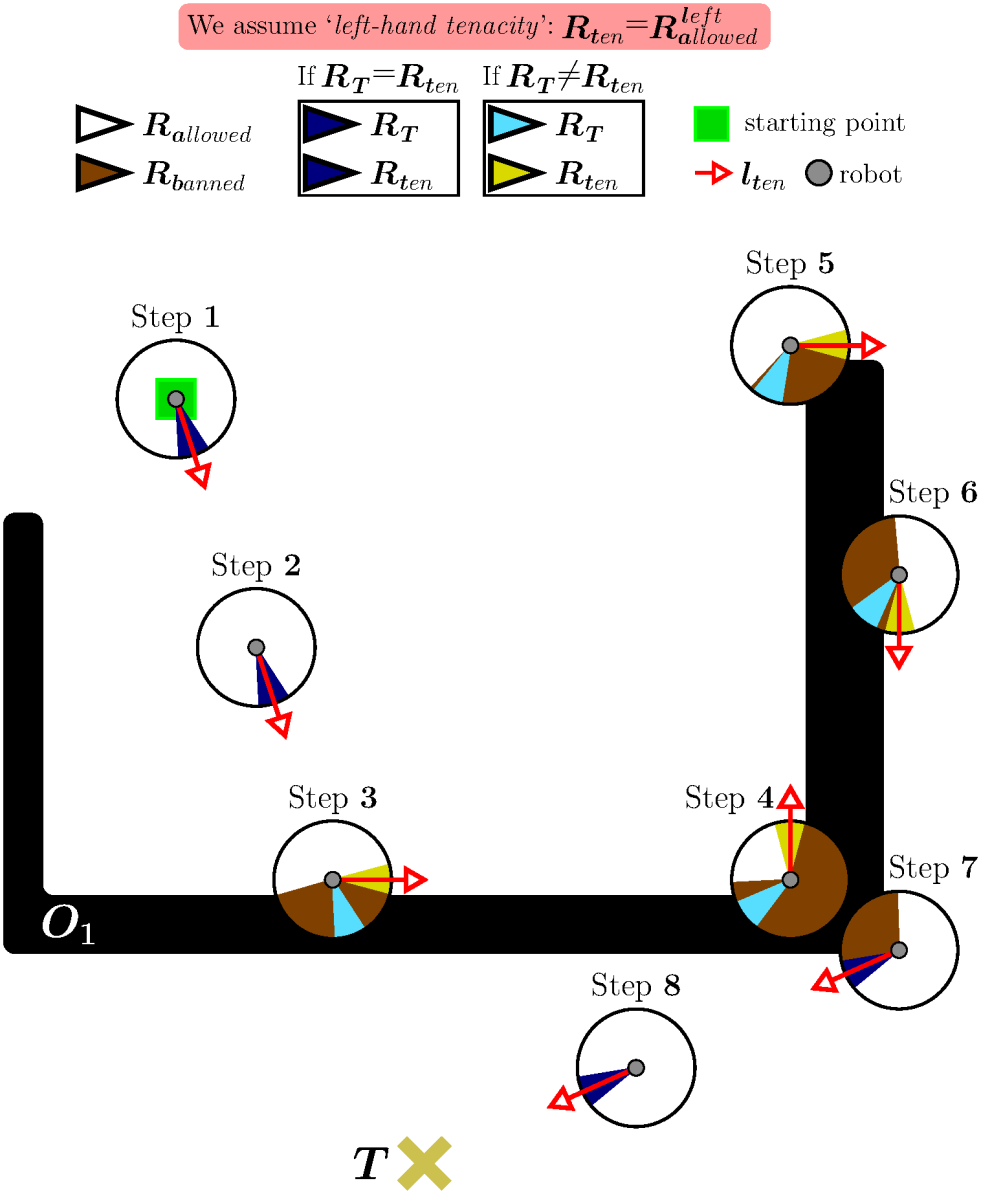
Behavior that emerges when the Traversability principle and the Tenacity principle are jointly applied.

Let us consider the two following assumptions: [*A_1_*] the principles of Traversability and Tenacity are integrated into the robot’s control loop (this fact actually means that these principles are applied again and again, at a sufficiently high rate, until the robot reaches *T*); and [*A_2_*] the robot moves along the straight line *l*_*ten*_. Under these assumptions, the behaviour of the robot does fit the pattern described below (in the following, *eBx* stands for “emerging Behavior number *x*”):

- *eB_1_*. The robot moves straight from its current position *C* to the desired target point *T*, as long as there is no obstacle in the path. This behaviour corresponds to steps 1 and 2 in Fig 5.
- *eB_2_*. When an obstacle prevents the robot from progressing towards *T*, the robot immediately switches to a boundary-following behaviour so as to circumvent the blocking obstacle.
  The robot follows the boundary of the obstacle in either clockwise or counterclockwise direction depending on the type of tenacity being used.
  The boundary-following behaviour explained above is that of steps 3 to 6 in Fig 5. In this figure, it is supposed that the principle of Tenacity is applied in its left-hand form—therefore, $R_{ten}=R_{allowed}^{left}$.
- *eB_3_*. Boundary-following continues until there is a clear and straight path to the target *T*, i.e. until *R*_*T*_ ∈ *S*_*allowed*_. This way of behaving corresponds to step 7 in Fig 5.

Finally, it is worth noting that, after eB_3_, the robot behaves according to eB_1_ again. Moreover, when this transition occurs, from eB_3_ to eB_1_, all data stored in the short-term memory are erased. For an illustration of these two final remarks, see step 8 in Fig 5.

#### Strengths and weaknesses

**Fig 6:**
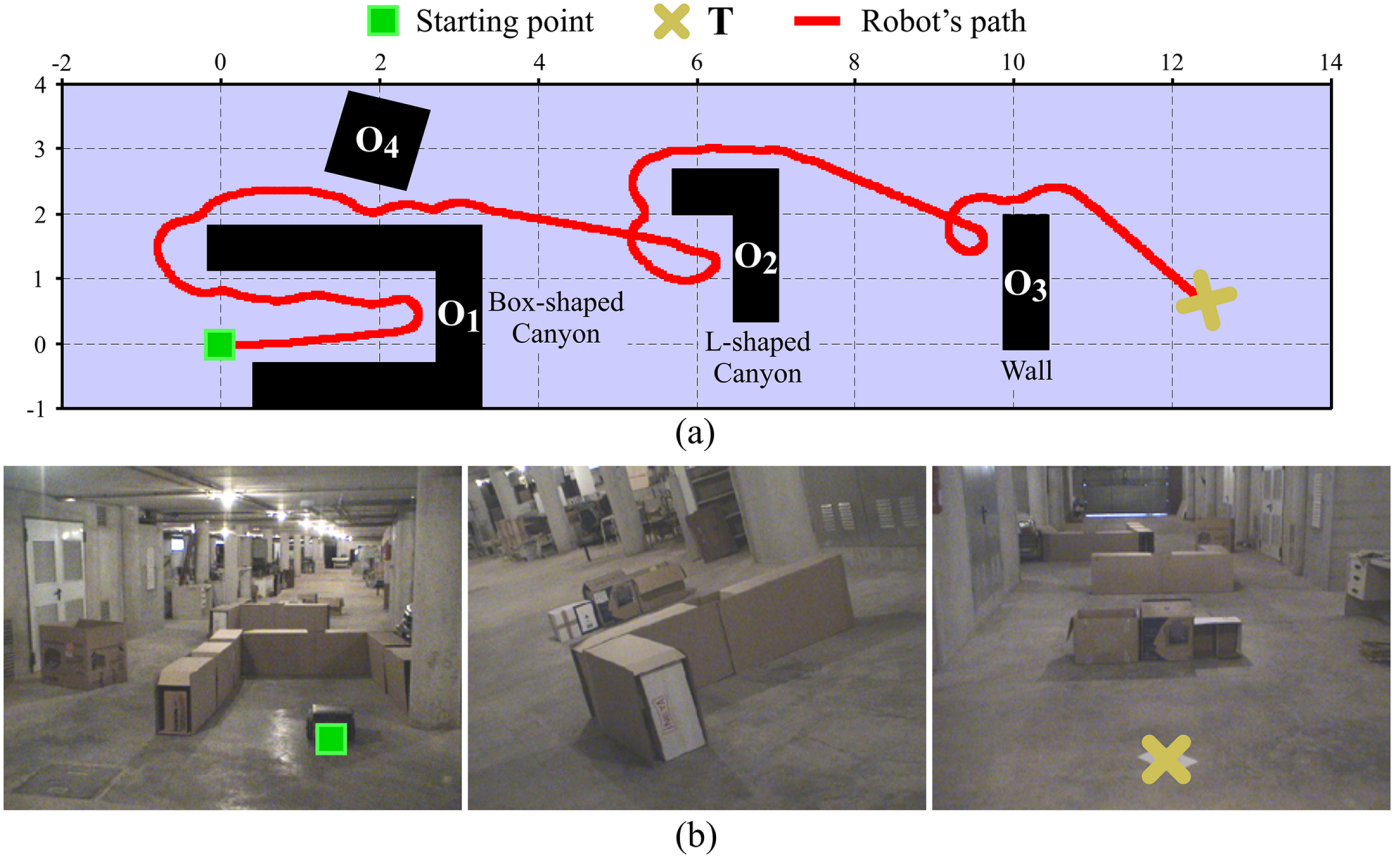
The T^2^ strategy escaping from several dead-end traps. The T^2^ strategy was configured in its left-hand tenacity mode. As can be seen, the robot needed to move away from the target in order to avoid the two canyon-like obstacles. (a) Plot of the path followed by the robot (over 28 meters in length). (b) Multiple views of the navigation environment.

Through extensive experimentation on both simulated and real robots, the T^2^ strategy has demonstrated to be able to make robots avoid very large and intricate obstacles, while successfully progressing towards the desired target point. As an example of this—extracted from [25]—, Fig 6 shows the trajectory generated by the T^2^ strategy in an environment with three main obstacles (the reader can find many more experiments in [25]). As can be observed, several cardboard boxes were strategically placed to build two canyons—one U-shaped and the other L-shaped—and a wall. The robot used in this experiment was a Pioneer 3-DX platform fitted with sixteen ultrasonic sensors, which were arranged to provide a 360-degree coverage. The sensing range of all these ultrasonic sensors was limited, by software, to just 0.75 metres. The reason behind this was to make the experiment more challenging, in the sense of ensuring that obstacles were never fully detected by the robot’s sensors (i.e. when an obstacle was detected by the ultrasonic sensors, some parts of it lied outside their maximum detection range).

**Fig 7:**
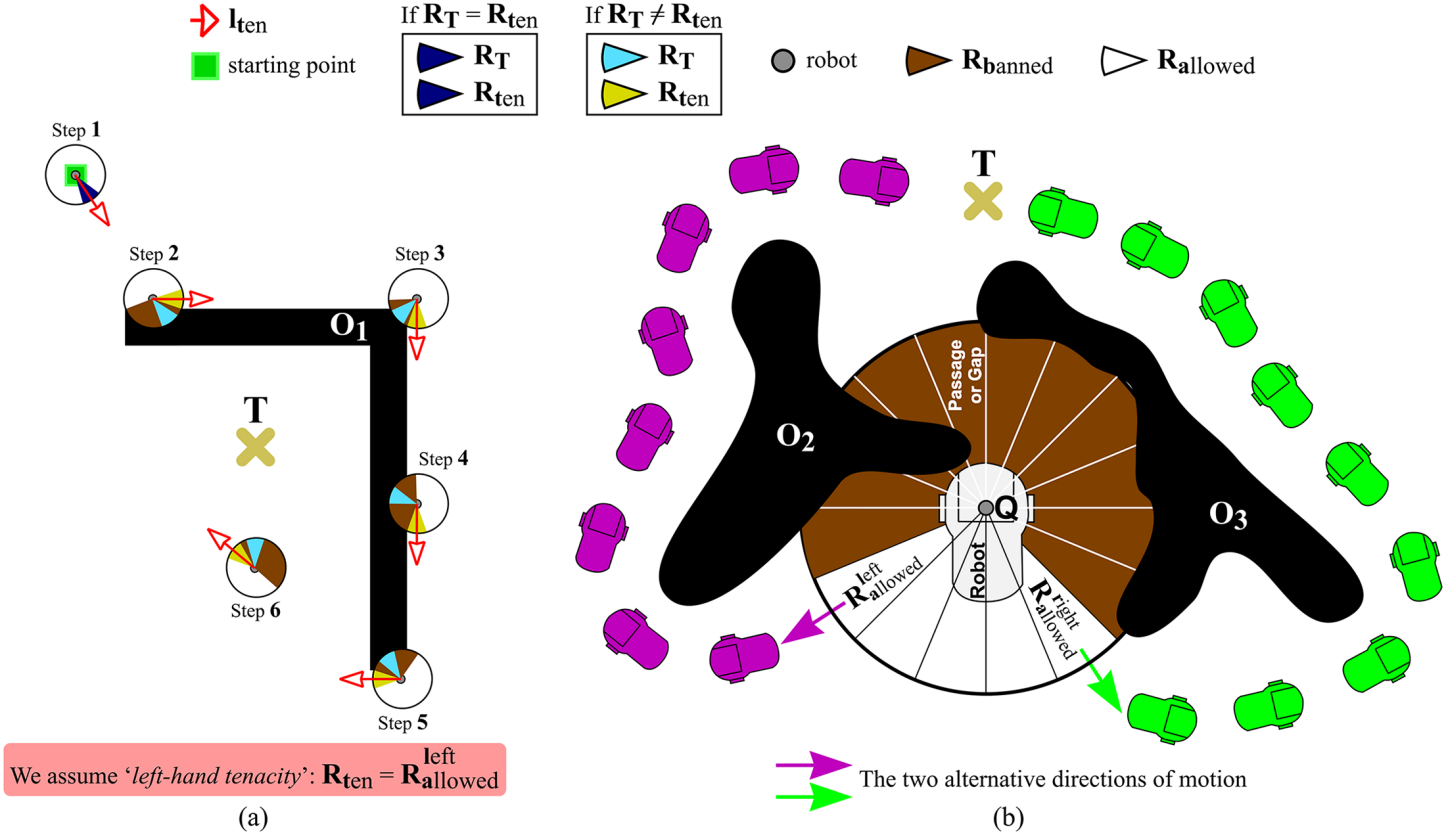
Some limitations of the T^2^ strategy. (a) The robot never converges to the target; (b) Narrow passages/gaps are missed (as a general remark, it is important to say that T^2^ inherently favors the navigation of the robot across the open spaces of the environment, where naturally there is less risk of collision).

Unfortunately, the T^2^ strategy also has serious limitations, which may cause either the robot never reaches the target point or the robot chooses a long path to get to it. We discuss next these limitations in detail (in the following, *Lx* stands for “Limitation number *x*”):

- *L1*. As a consequence of the use of the short-term memory—that is to say, as a consequence of remembering obstacles which were detected in the past (Fig 4(b))—, the T^2^ strategy may not allow the robot to directly progress towards the target point when no obstacle is really present between them.
  The situation illustrated in Fig 7(a) is an example of the aforementioned problem. As can be observed, after step 6, the robot is not allowed to head for the target because in the past it detected obstacles in that direction. Nevertheless, those obstacles are behind the target, and, therefore, they are not relevant at all for the navigation task. Despite this, in such a situation, the T^2^ strategy is unable to realize that the target is directly reachable by the robot.
- *L2*. The T^2^ strategy may fail to detect narrow passages/gaps between obstacles where the robot actually fits in. For some scenarios—such as the one of Fig 7(b)—, this gap-detection failure entails the loss of opportunities to reach the target point through a shorter path.
  As shown in Fig 7(b), none of the two possible motion directions computed by the T^2^ strategy at point *Q*—these directions are given by the $R_{allowed}^{right}$ and $R_{allowed}^{left}$ regions—move the robot towards the central passage, which represents the shortest way to the target. In the above scenario, the robot would follow the path marked in green if the T^2^ strategy was configured in its right-hand tenacity mode, or alternatively, the robot would follow the purple path if the left-hand tenacity mode was employed.

## *Escape Gap* as a new reactive control strategy

As shown in [28] and [25], TGF and T^2^ make the robot gain new capabilities/skills for navigation. To be precise, the former of these strategies does allow the robot to go through very narrow passages without collision (in this context, “very narrow” means just a few millimetres bigger than the size of the robot). On the other hand, with the T^2^ strategy, the robot does acquire the skill of being able to overcome large and complex obstacles (here: “large” means much larger in size than the area of sensing around the robot—as given by the maximum detection range of the robot’s sensors; and, by “complex”, we mean an obstacle that features twisting passages and dead-ends as well as places with only one way in and out).

As one can easily understand, both of the above-mentioned capabilities/skills are essential for any autonomous mobile robot which is intended to behave intelligently. In view thereof, it is clear that the combination of the skill provided by TGF and the skill provided by T^2^ is to provide enhanced navigation abilities to the robot. Consistently with this observation, this section proposes integrating TGF and T^2^ into a new reactive control strategy capable of manoeuvring a robot through tight passages and making a robot escape from huge maze-like obstacles while moving to the target *T* quickly. This new strategy goes under the name of *Escape Gap* or, simply, *EG*.

Below, we will start with a short description of EG. After that, we will discuss specific details about the operation of this new strategy.

### General overview of EG

**Fig 8:**
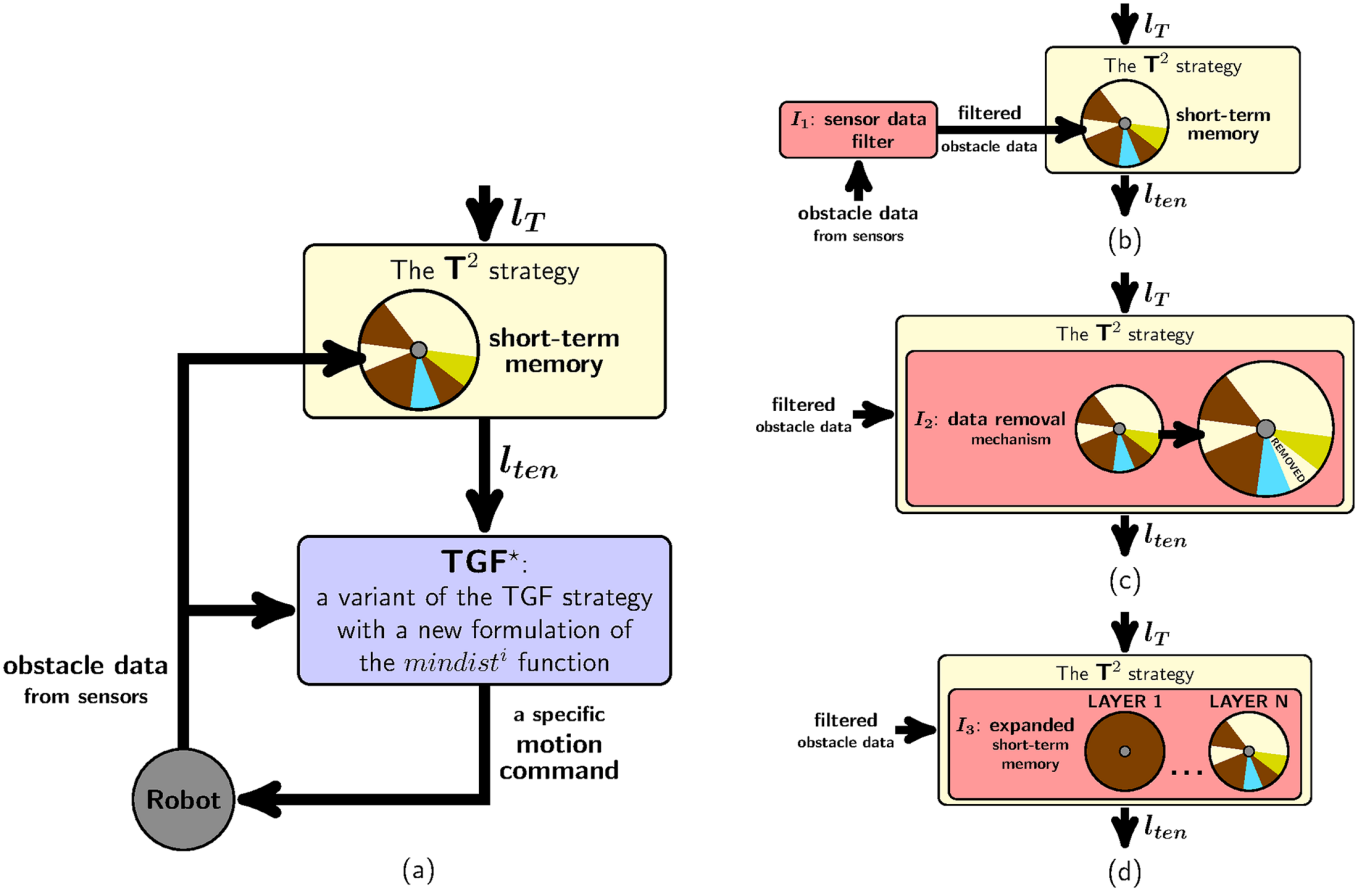
A schematic view of the components of EG. (a) first proposal; (b,c,d) some improvements to (a) (in these figures, *I*_*num*_ is used as a shorthand term of *Improvement #num*).

A block diagram of the EG strategy is sketched in Fig 8(a). As can be observed, only two blocks appear: one of them—the first block at the top—represents the T^2^ strategy as described in [25]; the other block represents a variant of the TGF strategy (as we will explain further on, the main difference between the TGF strategy presented in [28] and the one integrated into EG lies in how they do define the *mindist*^*i*^ function).

In order to work properly, the T^2^ strategy needs to know the final destination of the robot and the location of those obstacles which have recently been detected by the robot (as is clear from Fig 8(a), the information about the robot’s destination is passed through the input *l*_*T*_, while the information about the location of all recently detected obstacles is retrieved from an internal short-term memory which is continuously fed with sensory data). The two pieces of information listed above are used by the T^2^ strategy to compute the output *l*_*ten*_. Here, it is worth stepping back to remember that *l*_*ten*_ is a line which indicates a suitable direction of motion for the robot for the current time instant. By moving along *l*_*ten*_, the robot is expected to not get stuck against any obstacle; or, what is still more important, the robot is expected to succeed in finding a path to reach its final destination *T*.

In its original version [28], the TGF strategy requires two inputs: on the one hand, the first input is the final destination of the robot (just like the T^2^ strategy, TGF assumes that such a destination is determined by the straight-line segment *l*_*T*_); on the other hand, the second input corresponds to the location of all nearby obstacles (by the expression “nearby obstacles”, we mean the obstacles that are currently visible to the robot’s sensors). Using these two inputs, the TGF strategy chooses the gap through which the robot should pass, and generates a control command accordingly.

The TGF strategy integrated into EG differs from the original in two primary aspects: the value of one of their inputs and the internal definition of the *mindist*^*i*^ function (this function is of great importance, because it is in charge of choosing the best gap for the robot to go through; for the sake of clarity, from now on, we will refer to the original version of TGF as *TGF* and to the modified version of TGF—i.e. the one used in EG—as *TGF*^⋆^).

As for the first of the above differentiating aspects, both TGF and TGF^⋆^ have an input that allows them to know in which direction the target that the robot should reach lies. This input is what we have previously called “the destination of the robot”. As has been already explained, the destination of the robot is represented by *l*_*T*_ in TGF. Rather, in TGF^⋆^, such a destination is supposed to be given by *l*_*ten*_, as is evident from the fact that the output of the T^2^ strategy is connected to TGF^⋆^ in Fig 8(a) (plainly speaking, *l*_*ten*_ is a line which coincides with *l*_*T*_ when there are no obstacles to overcome; alternatively, when the robot faces an obstacle, *l*_*ten*_ points in the direction of the avoidance route to be followed).

The second differentiating aspect between TGF and TGF^⋆^ is the *mindist*^*i*^ function. In TGF, the expression for this function is the one of eq 1. By contrast, TGF^⋆^ defines the *mindist*^*i*^ function as follows (the reader is referred to the section where the TGF strategy was described for a definition of the terms $l_{1}^{i}$ and $l_{2}^{i}$, as well as the function *dist*):

$$\begin{array}{c} min(dist(l_{ten},l_{1}^{i}),dist(l_{ten},l_{2}^{i})). \end{array}$$ (4)

With this new expression of the *mindist*^*i*^ function, we cause TGF^⋆^ to drive the robot towards the gap which is closest to the direction suggested by the T^2^ strategy.

We conclude this section with an easy-to-understand way of interpreting Fig 8(a). With respect to this figure, we can say that: (*i*) the T^2^ component of EG provides a rough estimate of the free-obstacle path that the robot should traverse in order to reach the target *T*; (*ii*) the TGF^⋆^ component of EG takes the previous path, refines it—mainly in those parts where the path goes through narrow passages—, and, finally, makes the robot follow it. Here, it is of utmost importance to note that everything in (*i*) and (*ii*) is done in accordance with the spirit of the reactive paradigm, which means that EG—or, equivalently, its two components: T^2^ and TGF^⋆^—does work by means of only a partial/local view of the environment.

### Shortcomings and improvements of EG

The EG strategy, as has been stated in the previous section, makes the robot behave in an undesirable manner in some cases. Next, these cases are examined in detail, and an appropriate solution is given for each of them.

#### Case *A*: Some gaps are ignored

**Fig 9:**
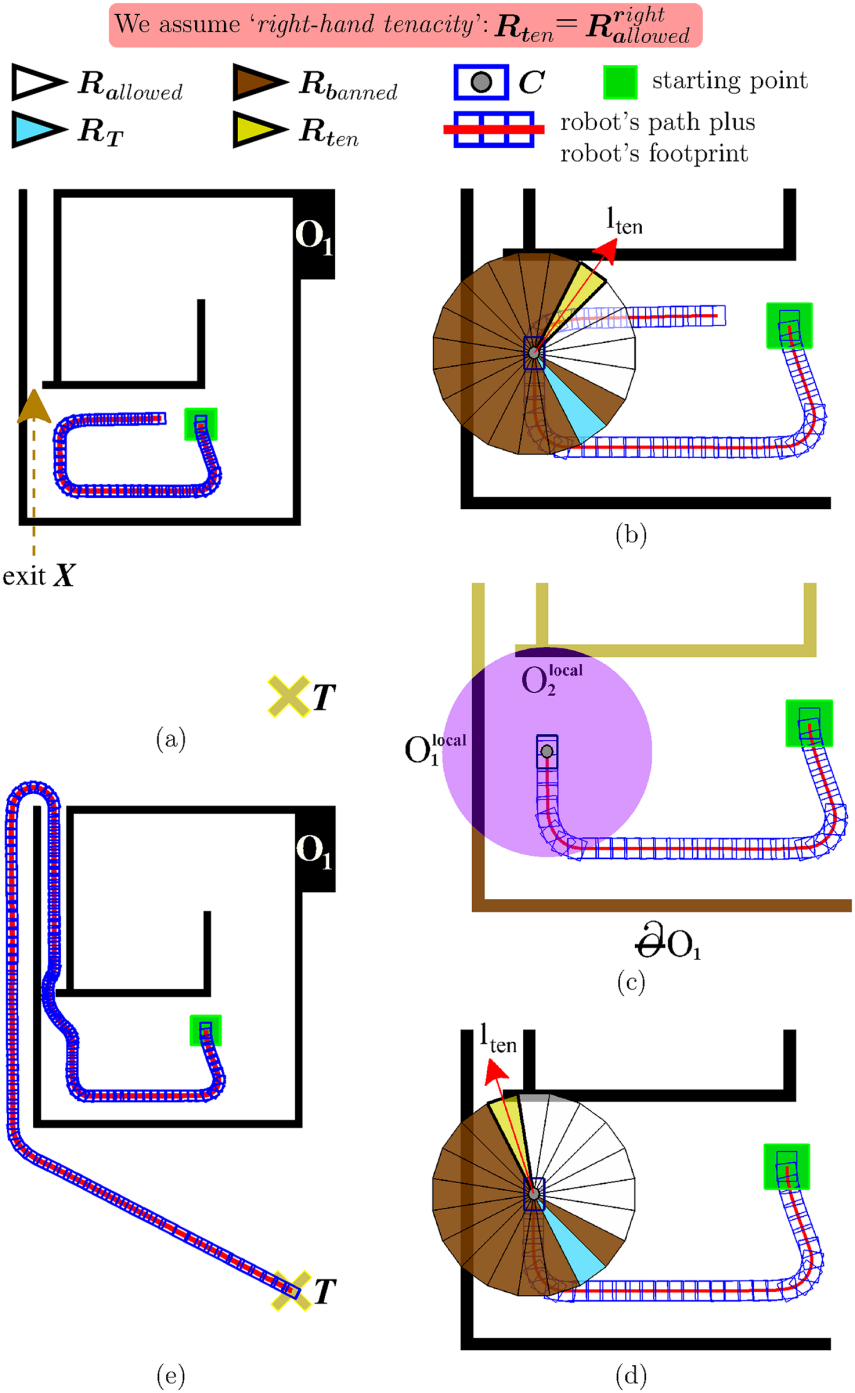
A comparison of the performance of EG. (a,b) before and (c,d,e) after adding the sensor data filter.

Fig 9(a) shows a first case where EG fails in guiding the robot to the desired target point *T*. From this figure, several observations can be made: first of all, the environment is such that the only way to escape from the interior of the obstacle *O*_1_ is by going through the exit marked with the dashed arrow (for simplicity, in the following lines, we will refer to this exit as *X*); on the other hand, taking a look at the trajectory followed by the robot—see the red line which is partly hidden by blue rectangles—, one can realize that the robot has passed very close to *X*, but has ignored it. Thus, a natural question arises: why has this happened? Fig 9(b) reveals that the above problem is due to an incorrect behaviour of the T^2^ component of EG: as can be appreciated, when the robot is in front of *X*, T^2^ suggests that the robot continues following the boundary of the obstacle *O*_1_ as if the exit *X* did not exist, and therefore moves away from *X*.

To solve the problem of Fig 9(b) (which is exactly the same problem as the one of Fig 7(b)), we propose to add a new sensor data filter into EG. Roughly speaking, such a filter builds on the idea that not all the information provided by the robot’s sensors should be stored in the short-term memory of the T^2^ strategy. To bring this idea to life, the sensor data filter has been connected to the other components of EG as illustrated in Fig 8(b). By connecting it this way, the sensor data filter acquires the power to decide which data, among those collected by the robot’s sensors, are—temporarily—memorized by T^2^ and which are not. Before elaborating on the details of the filtering criterion, we want to remark the following: (*i*) EG makes the robot adopt a boundary-following behaviour each time a blocking obstacle is found (this is a functionality inherited from the T^2^ strategy); (*ii*) the sensor data filter is active only when the robot is performing the aforesaid boundary-following behaviour (note additionally that all data are allowed to pass through the filter when it is not active); (*iii*) when we refer to “the data collected by the robot’s sensors”, we actually mean the data coming from the proximity sensors of the robot, i.e. the data concerning—nearby—obstacles. With remarks *i* to *iii* in mind, we are now in a position to describe the sensor data filter, which proceeds as follows: let *O*_*b*_ be the blocking obstacle that the robot is trying to avoid, and let ∂Ob be the portion of the *O*_*b*_’s boundary followed by the robot from the start of the boundary-following behaviour; then, *the sensor data filter does block all obstacles which are not contiguous to* ∂Ob.

To make things more clear, we give an example of how the sensor data filter works. To this end, we will use Fig 9(c), for which it is important to consider the following: the environment is the same as that of Fig 9(a) and 9(b) (there is just one obstacle named *O*_1_); the robot is assumed to be located at the same position as in Fig 9(b) (hereafter, we will refer to this position as *C*); the obstacles sensed by the robot at position *C* are all displayed in dark-violet colour (these obstacles are labelled using the notation $O_{n}^{local}$, where *n* is a sequential number); the rest of the obstacles—i.e. those outside the robot’s sensing area—are displayed in brown colour and in greenish-yellow colour; finally, the brown colour is also used to represent the portion of the *O*_1_’s boundary followed by the robot from the starting point to *C* (hereafter, we will refer to this portion as ∂O1). In the situation of Fig 9(c), the sensor data filter would work as follows: once the obstacles $O_{1}^{local}$ and $O_{2}^{local}$ were received as input, the filter would block obstacle $O_{2}^{local}$. The reason to do that is because obstacle $O_{1}^{local}$ extends ∂O1 by one of its ends, while obstacle $O_{2}^{local}$ does not. Now, to complete the above discussion, let us say a few words about the implications that the blocking of obstacle $O_{2}^{local}$ would have on navigation: at position *C*, the T^2^ strategy would advise the robot to move directly towards the exit *X* (see the pointing direction of *l*_*ten*_ in Fig 9(d)); after taking this exit, the robot would be capable of reaching *T*, as shown in Fig 9(e).

#### Case *B*: No progress to *T*

Just like the T^2^ strategy, EG is also susceptible to the problem of Fig 7(a). This situation shows that, occasionally, remembering the past—i.e. remember the position of the obstacles which were previously detected by the robot’s sensors—may prevent the robot from progressing to the target point, when there is no obstacle blocking the straight-line path that joins the robot and the target.

With the above in mind, it is clear that, for solving the problem highlighted in Fig 7(a), EG should forget some information from the past, specifically that information which is no longer necessary for the task of obstacle avoidance. In order to make this happen, a data removal mechanism has been added to EG, as reflected in Fig 8(c). In essence, such a data removal mechanism searches for inconsistencies between the information which is currently stored in the short-term memory and the information which is currently provided by the robot’s sensors. For the data removal mechanism, the term inconsistency exclusively refers to the fact that: the short-term memory indicates that there are obstacles in a certain region (*R*_0_…*R*_*K*−1_), but the sensors of the robot say just the opposite. When an inconsistency is found, the data removal mechanism removes those data from the short-term memory which are causing the inconsistency.

**Fig 10:**
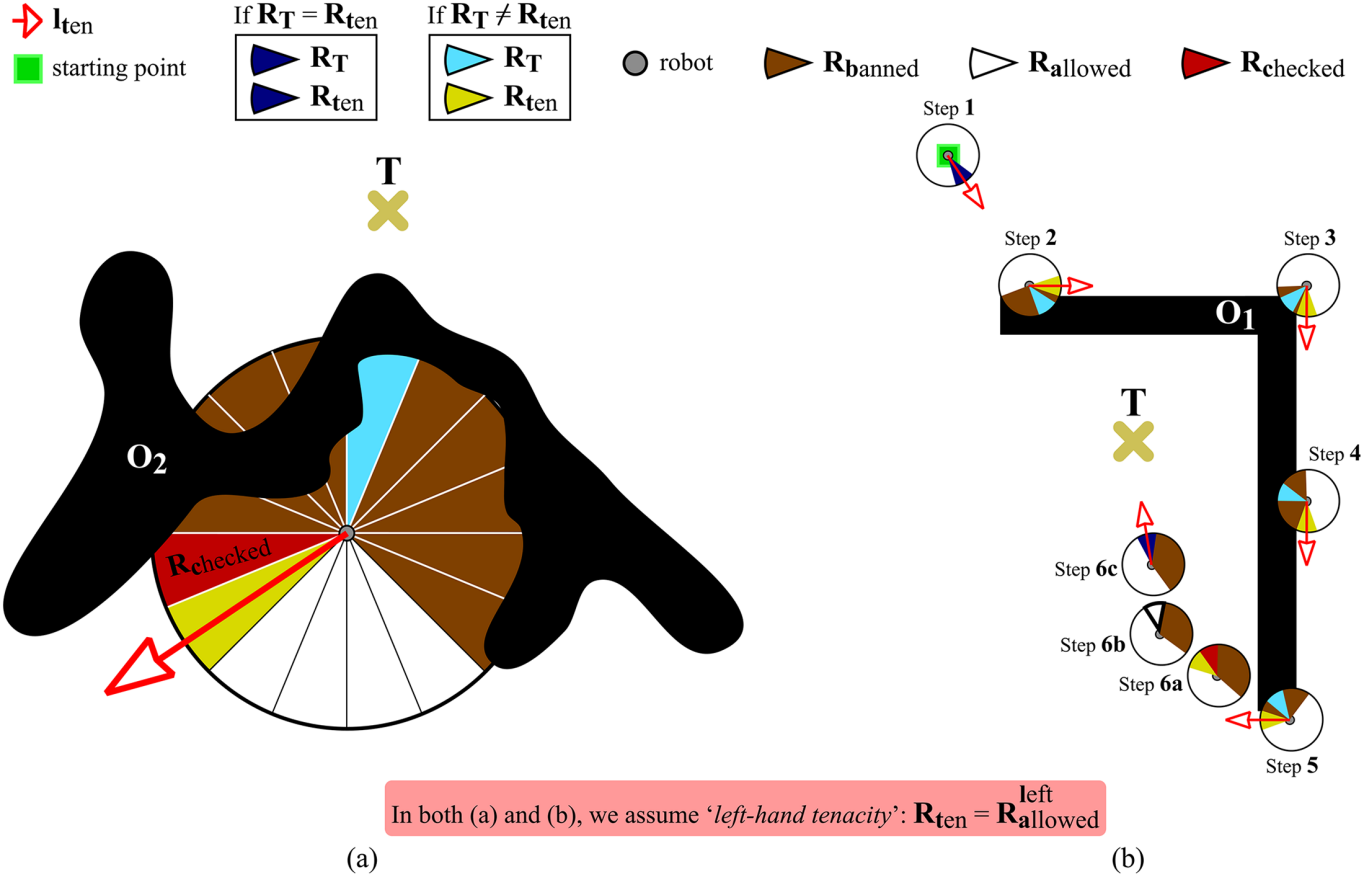
Removing the information about obstacles from the short-term memory which unnecessarily restricts the motion of the robot. (a) A graphical representation of what *R*_*checked*_ means. (b) The data removal mechanism in action: at step 6a, the *R*_*checked*_ region is considered to be inconsistent; accordingly, the *R*_*checked*_ region is relabelled as allowed at step 6b; at step 6c, the robot goes directly towards *T*.

Finally, it should be noted that the search for inconsistencies performed by the data removal mechanism is restricted to a small area of the short-term memory. Before going any further on this point, let us define *R*_*checked*_ as the banned-labelled region that is closest to *R*_*ten*_ (look at Fig 10(a)). According to this, we can state that the data removal mechanism only checks whether the *R*_*checked*_ region is inconsistent or not, in the sense given above. In case *R*_*checked*_ is inconsistent, its state will be changed to allowed. For the same environment as in Figs 7(a) and 10(b) depicts the path the robot would follow after incorporating our data removal mechanism.

#### Case *C*: Inability to escape from obstacles with multiple loops

**Fig 11:**
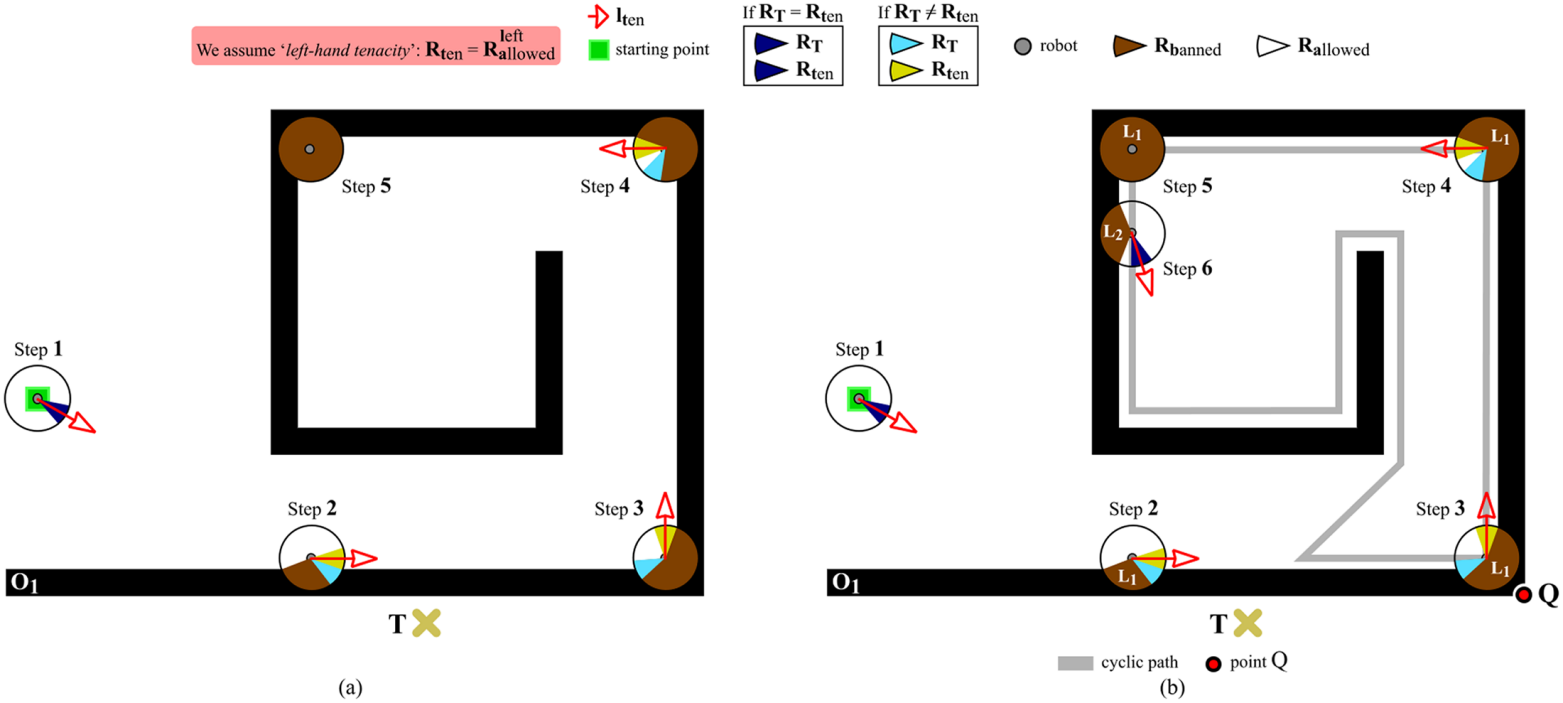
Problems and solutions for successfully avoiding spiral-like obstacles. (a) At step 5, the robot does not know where to go. (b) At step 6, a new layer is added to the short-term memory (in the figure, *L*_*i*_ stands for layer #i).

Let us imagine an environment where there is an obstacle with a spiral-like shape, such as the one of Fig 11(a). In this environment, EG is unable to compute *l*_*ten*_ at step 5, because there are no regions labelled as allowed (*S*_*allowed*_ = ∅). Consequently, from step 5 onwards, EG does not know in which direction the robot should move in order to reach the target point *T*.

To solve the problem described above, we propose to introduce the concept of *layer* into the short-term memory of EG. As diagrammed in Fig 8(d), the short-term memory may now comprise several *layers*. Briefly speaking, a layer is the compact view of the surroundings of the robot which was inherited from the T^2^ strategy (remember that this compact view consists of a 360-degree circle which is divided into *K* angular regions (*R*_0_ … *R*_*K*−1_), where each angular region can be labelled as either banned or allowed, depending on whether or not the robot has recently detected obstacles in the range of directions covered by the angular region—see Fig 4). Our solution to the problem of Fig 11(a) is to convert EG into a strategy able to support multiple layers organized as a LIFO memory.

When the navigation task starts, EG merely has one layer available (hereinafter, this layer will be indistinctly referred to as either main layer or layer #1). New layers are created according to the navigation needs. To be more precise, a second layer will be created when layer #1 becomes fully banned,—or, said differently, when the situation of step 5 in Fig 11(a) happens. In more general and formal words, we could state that: let layer #i be the last layer which has been created, and let $S_{allowed}^{i}$ denote the set of all angular regions labelled as allowed in layer #i; then, the layer #i+1 will be created if and only if $S_{allowed}^{i}=\emptyset$. When a layer is created, it immediately turns into the currently-active layer (by default, the currently-active layer is layer #1; no more than one layer may be currently-active, which means that: if layer #i+1 is created, it will become the currently-active layer and layer #i will stop being currently-active). At this point, it should be noted that the information captured by the robot’s sensors is only incorporated into the currently-active layer (the rest of layers do not receive any information). Fig 11(b) shows an example of creation of a new layer. As can be seen, from steps 1 to 5, layer #1 is the currently-active layer. At step 5, the above-stated condition for layer creation is met. In consequence, layer #2 is created at step 6. From that moment on, layer #2 will be the currently-active layer and will receive as input all the data coming from the robot’s sensors.

By analyzing Fig 11(b) in detail, one can quickly realize that a new problem arises at step 6. On the one hand, in accordance with eqs 2 and 3, $R_{allowed}^{left}=R_{allowed}^{right}=R_{T}$. On the other hand, taking into account the assumption made by Fig 11 of using EG in its left-hand tenacity mode, $R_{ten}=R_{allowed}^{left}=R_{T}$. Practically speaking, this means that the robot will stop following the boundary of the *O*_1_ obstacle and will move directly towards the target point, *T*. However, this is not the best thing the robot can do in such a situation. Cyclic behaviors may occur if the robot leaves off doing boundary-following in an inner loop of a spiral-like obstacle. By way of illustration, the robot would perform the cyclic path in gray of Fig 11(b), if EG would decide to leave the *O*_1_’s boundary at step 6. Our objective is to make the robot continue following the *O*_1_’s boundary until point *Q* of Fig 11(b), because this point ensures the definitive avoidance of the *O*_1_ obstacle. To attain our objective, we have to change the way how EG computes $R_{allowed}^{right}$ and $R_{allowed}^{left}$. In view of that, we propose to rewrite eqs 2 as 5 and 3 as 6.

$$\begin{array}{c} R_{allowed}^{j,right}=\{\begin{array}{cc} \text{if}\;{\text{MAINCND}}^{j}, & R_{allowed}^{j,right}=R_{T}^{j}\\ \text{if}\;\neg {\text{MAINCND}}^{j}\;\text{and}\;R_{T}^{j}\in S_{banned}^{j}, & R_{allowed}^{j,right}\;\text{is}\;\text{the}\;\text{angular}\;\text{region}\\ & \text{of}\;S_{allowed}^{j}\;\text{that}\;\text{is}\;\text{closest}\;\text{to}\\ & R_{T}^{j}\;\text{clockwise}\\ \text{otherwise}, & R_{allowed}^{j,right}\;\text{is}\;\text{the}\;\text{angular}\;\text{region}\\ & \text{of}\;S_{allowed}^{j}\;\text{that}\;\text{is}\;\text{closest}\;\text{to}\\ & R_{banned}^{j,right}\;\text{clockwise}. \end{array} \end{array}$$ (5)

$$\begin{array}{c} R_{allowed}^{j,left}=\{\begin{array}{cc} \text{if}\;{\text{MAINCND}}^{j}, & R_{allowed}^{j,left}=R_{T}^{j}\\ \text{if}\;\neg {\text{MAINCND}}^{j}\;\text{and}\;R_{T}^{j}\in S_{banned}^{j}, & R_{allowed}^{j,left}\;\text{is}\;\text{the}\;\text{angular}\;\text{region}\\ & \text{of}\;S_{allowed}^{j}\;\text{that}\;\text{is}\;\text{closest}\;\text{to}\\ & R_{T}^{j}\;\text{counterclockwise}\\ \text{otherwise}, & R_{allowed}^{j,left}\;\text{is}\;\text{the}\;\text{angular}\;\text{region}\\ & \text{of}\;S_{allowed}^{j}\;\text{that}\;\text{is}\;\text{closest}\;\text{to}\\ & R_{banned}^{j,left}\;\text{counterclockwise}. \end{array} \end{array}$$ (6)

Before discussing the meaning of eqs 5 and 6, let us introduce the notation employed in both equations. In short: $L_{CA}^{j}$ denotes the layer which is currently-active at step *j*; *S*^*j*^ refers to the set of all angular regions of $L_{CA}^{j}$; $S_{allowed}^{j}$ is the subset of *S*^*j*^ that contains all angular regions labelled as allowed; $S_{banned}^{j}$ is the subset of *S*^*j*^ that contains all angular regions labelled as banned; *C*^*j*^ represents the position of the robot at step *j*; $l_{T}^{j}$ denotes the straight-line segment that joins *C*^*j*^ to *T*; $R_{T}^{j}$ designates the angular region of *S*^*j*^ in which $l_{T}^{j}$ falls; $R_{banned}^{j,right}$ is the angular region of $S_{banned}^{j}$ that is closest to $R_{T}^{j}$ counterclockwise; $R_{banned}^{j,left}$ is the angular region of $S_{banned}^{j}$ that is closest to $R_{T}^{j}$ clockwise; and, finally, the so-called MAINCND^*j*^ condition is defined by eq 7.

$$\begin{array}{c} \begin{array}{cc} {\text{MAINCND}}^{j}= & \;“\text{a}\;\text{new}\;\text{layer}\;\text{is}\;\text{not}\;\text{created}\;\text{at}\;\text{step}\;\text{j}”\;\text{and}\\ & \;R_{T}^{j-1}\in S_{banned}^{j-1}\;\text{and}\;R_{T}^{j}\in S_{allowed}^{j} \end{array} \end{array}$$ (7)

**Fig 12:**
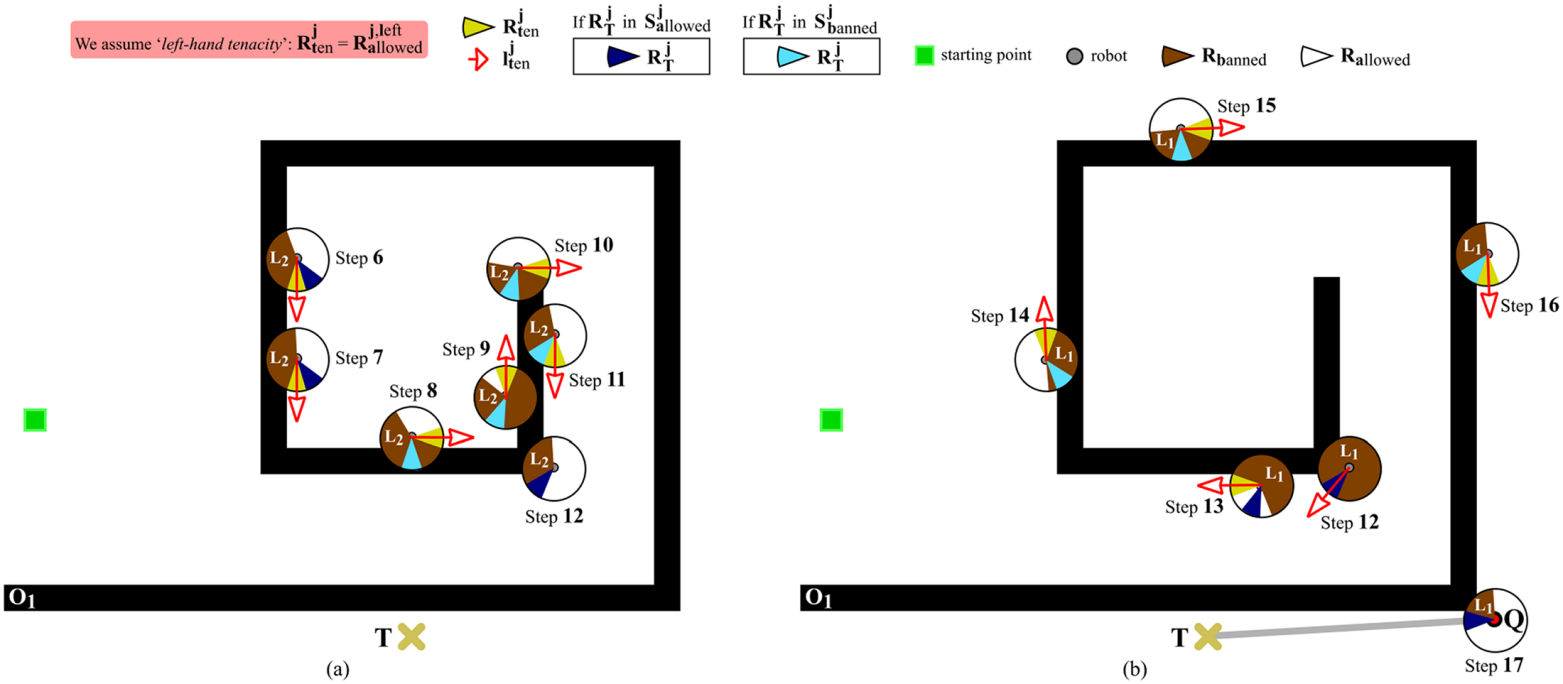
Continuation of the navigation task initiated in Fig 11(a) and 11(b). The situation depicted makes clear that EG allows the robot to progress directly towards the target point (meanwhile, the robot persists in performing boundary-following) just when the robot reaches the outermost loop of the spiral-like obstacle and the currently-active layer of the short-term memory indicates that there is a free-obstacle path to *T*. (a) At step 12, layer #2 is destroyed, and layer #1 is recovered and used for deciding where to move the robot. (b) At step 17, the robot leaves the *O*_1_’s boundary and moves following the thick gray line. In both figures, (a) and (b), *L*_*i*_ stands for layer #i. Lastly, it is also important to note that the legend of this figure gives a different meaning to some colors as compared to the legend of Fig 11(a) and 11(b). Essentially, now the $R_{T}^{j}$ angular region is painted in either dark-blue or soft-blue depending on how it is labelled: dark-blue/soft-blue is used when $R_{T}^{j}$ is labelled as allowed/banned.

**Fig 13:**
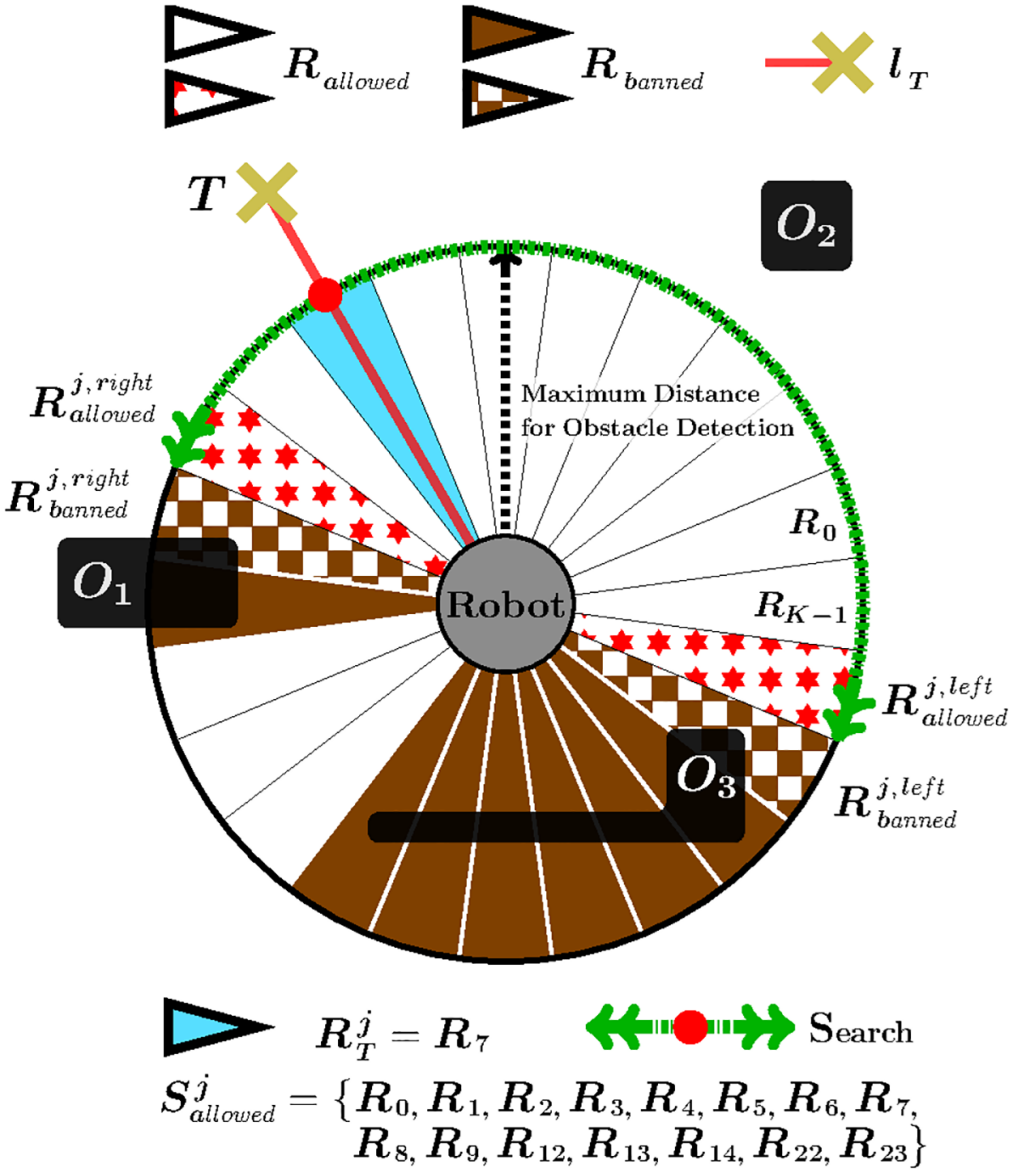
One of the three different ways of calculating $R_{allowed}^{j,right}$ and $R_{allowed}^{j,left}$ that eqs 5 and 6 do propose. The use of this way of calculating $R_{allowed}^{j,right}$ and $R_{allowed}^{j,left}$ is restricted to the case in which the robot is navigating inside a spiral-like obstacle and the EG’s short-term memory detects a *false* free-obstacle path towards the target point.

We now make use of Fig 12 to explain, in clear and practical terms, eq 6 (the forthcoming discussion can be easily extrapolated to the case of eq 5). This figure assumes that the robot is at step 6 of Fig 11(b). Next, we will see that, by means of eq 6, EG ensures the robot keeps on doing its boundary-following behavior until reaching point *Q*. Let us analyze Fig 12(a) and 12(b) step by step:

Step 6 As has been mentioned before, EG creates layer #2 at step 6. As a consequence of this, the MAINCND^*j* = 6^ condition is not satisfied (see eq 7). Additionally, $R_{T}^{j=6}$ is an allowed-labelled region. Therefore, taking into consideration all the above ($\neg {\text{MAINCND}}^{j=6}\;\text{and}\;R_{T}^{j=6}\in S_{allowed}^{j=6}$), $R_{allowed}^{j=6,left}$ is calculated using the *otherwise* part of eq 6. This part of eq 6 suggests computing $R_{allowed}^{j=6,left}$ as illustrated in Fig 13. Plainly speaking, here EG forces the robot to continue following the *O*_1_’s boundary, despite there is an apparent free-obstacle path to *T* (as indicated by the fact that $R_{T}^{j=6}\in S_{allowed}^{j=6}$).
Step 7 This step is very similar to step 6, since the same condition holds: $\neg {\text{MAINCND}}^{j=7}\;\text{and}\;R_{T}^{j=7}\in S_{allowed}^{j=7}$. The only difference with respect to step 6 is the reason why MAINCND^*j* = 7^ is not satisfied. To be precise, MAINCND^*j* = 7^ is not met because $R_{T}^{6}$ is an allowed-labelled region, which is contrary to $R_{T}^{j-1=6}\in S_{banned}^{j-1=6}$ (refer to eq 7 again).
  As a conclusion of step 7, we can say that $R_{allowed}^{j=7,left}$ is obtained according to the *otherwise* part of eq 6, as was the case at step 6.
Step 8 Let us take a step back to remember that $R_{T}^{7}$ is an allowed-labelled region. At step 8, the above implies the non-fulfillment of the MAINCND^*j* = 8^ condition (note that $R_{T}^{j-1=7}\in S_{banned}^{j-1=7}$ is not true). In addition, $R_{T}^{j=8}$ is a banned-labelled region. All this means that: $\neg {\text{MAINCND}}^{j=8}\;\text{and}\;R_{T}^{j=8}\in S_{banned}^{j=8}$. Therefore, $R_{allowed}^{j=8,left}$ is calculated through the second part of eq 6. This part of eq 6 computes $R_{allowed}^{j=8,left}$ as explained earlier in Fig 4(a). Plainly speaking, here EG does not detect any free-obstacle path to *T* ($R_{T}^{j=8}\in S_{banned}^{j=8}$), and, in consequence, EG has no choice but to make the robot continue following the *O*_1_’s boundary.
Step 9 This step is very similar to step 8, since the same condition holds: $\neg {\text{MAINCND}}^{j=9}\;\text{and}\;R_{T}^{j=9}\in S_{banned}^{j=9}$. The only difference regarding step 8 is the reason why MAINCND^*j* = 9^ is not satisfied. To be precise, MAINCND^*j* = 9^ is not met because $R_{T}^{j=9}$ is a banned-labelled region, which goes against the condition imposed by eq 7 that $R_{T}^{j=9}\in S_{allowed}^{j=9}$. In short, $R_{allowed}^{j=9,left}$ is obtained in the same way as $R_{allowed}^{j=8,left}$, i.e. by applying the second part of eq 6.
  Next, steps 10 and 11 are omitted because they are identical to step 9.
Step 12 Here, we have the first step where the MAINCND^*j*=12^ condition is satisfied (by observing Fig 12(a), one can realize that the three conditions of eq 7 are actually met: on the one hand, $S_{allowed}^{j=12}$ is not an empty set and, in consequence, EG does not need to create a new layer at step 12; on the other hand, $R_{T}^{j-1=11}\in S_{banned}^{j-1=11}$; and, finally, $R_{T}^{j=12}\in S_{allowed}^{j=12}$). When the above happens, a set of actions is carried out: (1) layer #2—i.e. the currently-active layer—is destroyed (each layer stores information about the obstacles that the robot has found while following the boundary of a certain loop of the spiral-like obstacle; or in other words, layer #i contains information related to the i-th loop of the spiral-like obstacle, being the 1-st loop the outermost one; when the robot gets to circumnavigate the i-th loop of the spiral-like obstacle, EG decides to destroy layer #i since the information that this layer has is no longer necessary—besides, in this way, the use of memory is reduced); (2) layer #1 is turns into the currently-active layer (through this action, EG recovers all the information regarding the next loop of the spiral-like obstacle); and, as a last action, (3) $R_{allowed}^{j=12,left}$ is set to $R_{T}^{j=12}$ in conformity with the first part of eq 6 (in this respect, several observations should be made: first of all, the reason why the first part of eq 6 is applied is that MAINCND^*j*=12^ holds; and, secondly, when we do $R_{allowed}^{j=12,left}=R_{T}^{j=12}$, we are supposing that $R_{T}^{j=12}$ is an allowed-labelled region; however, this is not true since action (2) recovered layer #1 as it appears at step 5 of Fig 11(a), and, therefore, all its regions are banned-labelled; in order to solve this problem, $R_{T}^{j=12}$ is re-labelled as allowed, as shown by Fig 12(b)—look just at step 12.
Step 13 There are no differences between this step and step 7. One noteworthy aspect of step 13 is that more regions have been labelled as allowed in comparison with step 12. This is caused by the data removal mechanism which was previously added to EG—see Fig 8(c).
  Some of the following steps are identical to others which have already been discussed. More specifically: step 14 is equivalent to step 8; and, steps 15 and 16 are the same as steps 9, 10 and 11.
Step 17 At this step, the MAINCND^*j*=17^ condition is satisfied again. When this occurs being layer #1 the currently-active layer, some special actions are taken: (1) layer #1 is emptied, i.e. all data in it are deleted (note that layer #1 is not destroyed because it is the default layer of the short-term memory); (2) EG makes the robot stop following the *O*_1_’s boundary (this is because EG knows that the *O*_1_ obstacle has been definitively avoided); and, to finish, (3) EG moves the robot along a straight-line path to *T*, as evidenced by the thick gray line of Fig 12(b).

### EG in pseudocode

This section provides a comprehensive description in pseudocode of the EG strategy. Specifically, algorithm 1 shows how EG calculates $R_{ten}^{j}$. Recall that $R_{ten}^{j}$ represents the angular region which is selected after applying the principles of Traversability and Tenacity. Or said differently, $R_{ten}^{j}=R_{allowed}^{j,left}$ when the principle of Tenacity is applied in its left-hand form, and $R_{ten}^{j}=R_{allowed}^{j,right}$ when the principle of Tenacity is applied in its right-hand form.

To implement algorithm 1, several auxiliary functions and procedures have been used. All these functions and procedures are described in algorithm 2, and Tables 3 and 4.

**Algorithm 1** Calculation of *R*_*ten*_ at step *j* ($R_{ten}^{j}$)

1: **function** CALCRJTEN($R_{T}^{j-1},l_{T}^{j},toTenacity$)

 **INPUT PARAMETERS:**

  • $R_{T}^{j-1}$ is the value computed in line 5 of the CALCRJTEN function at the previous step *j* − 1

  • $l_{T}^{j}$ is the line joining the position of the robot at step *j* and the target point *T*

  • *toTenacity* determines the type of tenacity to be applied (possible values are either LEFT or RIGHT)

    ⊳ Lines 2 to 4 implement the functionality of the **sensor data filter**

2:   *rawSensorData* ← *readSensorData*();

3:   $filteredSensorData\leftarrow filterSensorData(L_{CA}^{j},rawSensorData);$

4:   $addDataToSTM(L_{CA}^{j},filteredSensorData);$

    ⊳ Lines 5 to 12 make the robot **move straight towards the target**

5:   $R_{T}^{j}\leftarrow computeRt\left(L_{CA}^{j},l_{T}^{j}\right);$

6:   **if** *getMode*() == *MtT* **then**

7:    **if** $isAllowed(R_{T}^{j})$ **then**

8:     $R_{ten}^{j}\leftarrow R_{T}^{j};$

9:    **else**

10:     *setMode*(*BF*);

11:    **end if**

12:   **end if**

   ⊳ Lines 13 to 15 make the robot **follow the boundary of a blocking obstacle**

13:   **if** *getMode*() == *BF* **then**

14:    $R_{ten}^{j}$ ← FLWBOUNDARY($R_{T}^{j-1},l_{T}^{j},toTenacity,filteredSensorData$);

15:    ⊳ See algorithm 2

16:   **end if**

17:   **return** $R_{ten}^{j};$

18: **end function**

**Algorithm 2** Auxiliary function that calculates $R_{ten}^{j}$ when the robot is in *BF* mode

1: **function** FLWBOUNDARY($R_{T}^{j-1},l_{T}^{j},toTenacity,filteredSensorData$)

 **INPUT PARAMETERS:**

  • The first three parameters are exactly the same as for CALCRJTEN

  • *filteredSensorData* are the data sensed by the robot at step *j* after the filtering step applied by the sensor data filter

    ⊳ Lines 2 to 31 allow the short-term memory to support **multiple layers**

            ⊳ Lines 2 to 8 control the **creation of a new layer**

2:   **if** $\left(isFullyBanned\left(L_{CA}^{j}\right)==true\right)$ **then**

3:    $L_{CA}^{j}\leftarrow createLayerInSTM\left(\right);$

4:    $addDataToSTM(L_{CA}^{j},filteredSensorData);$

5:    *newLayer* ← *true*;

6:   **else**

7:    *newLayer* ← *false*;

8:   **end if**

 ⊳ Lines 9 to 31 **solve** eq 5 when toTenacity is RIGHT **and** eq 6 when toTenacity is LEFT. In addition, note that the MAINCND^j^ condition is evaluated in line 17

9:   $R_{T}^{j}\leftarrow computeRt\left(L_{CA}^{j},l_{T}^{j}\right);$

10:   **if** *toTenacity* == *RIGHT* **then**

11:    *toSearchA* ← *clockwise*;

12:    *toSearchB* ← *counterclockwise*;

13:   **else**

14:    *toSearchA* ← *counterclockwise*;

15:    *toSearchB* ← *clockwise*;

16:   **end if**

17:   **if** $newLayer==false\&isBanned\left(R_{T}^{j-1}\right)\&isAllowed\left(R_{T}^{j}\right)$ **then**

18:    **if** $isLayer\#1(L_{CA}^{j})==false$ **then**

19:     $L_{CA}^{j}\leftarrow destroyLayerInSTM\left(\right);$

20:     $removeDataFromSTM(L_{CA}^{j},R_{T}^{j});$

21:    **else**

22:     $removeAllDataFromSTM(L_{CA}^{j});$

23:     *setMode*(*MtT*);

24:    **end if**

25:    $R_{ten}^{j}\leftarrow R_{T}^{j};$

26:   **else if** $isBanned(R_{T}^{j})$ **then**

27:    $R_{ten}^{j}\leftarrow getAllowedRegionClosestTo\left(L_{CA}^{j},R_{T}^{j},toSearchA\right);$

28:   **else**

29:    $R_{banned}^{j}\leftarrow getBannedRegionClosestTo\left(L_{CA}^{j},R_{T}^{j},toSearchB\right);$

30:    $R_{ten}^{j}\leftarrow getAllowedRegionClosestTo\left(L_{CA}^{j},R_{banned}^{j},toSearchA\right);$

31:   **end if**

    ⊳ Lines 32 to 36 implement the functionality of the **data removal mechanism**

32:   $R_{checked}\leftarrow getBannedRegionClosestTo\left(L_{CA}^{j},R_{ten}^{j},toSearchB\right);$

33:   **if** (*isInconsistent*(*R*_*checked*_, *filteredSensorData*) == *true*) **then**

34:    $removeDataFromSTM(L_{CA}^{j},R_{checked});$

35:    $R_{ten}^{j}\leftarrow R_{checked};$

36:   **end if**

37:   **return** $R_{ten}^{j};$

38: **end function**

**Table 3:** A brief description of the auxiliary functions and procedures used in algorithms 1 and 2.

Name	Description
**NOTES:** *STM* stands for Short-Term Memory *MtT* stands for Motion to Target *BF* stands for Boundary Following	
*readSensorData*()	This function reads the data provided by the obstacle detection sensors attached to the robot.
*filterSensorData*(*p*_*a*_, *p*_*b*_)	This function returns a subset of the data contained in *p*_*b*_. To be more precise, this function implements the functionality of the sensor data filter; consequently, it returns those data in *p*_*b*_ which are contiguous to the data stored in the STM layer *p*_*a*_.
*addDataToSTM*(*p*_*a*_, *p*_*b*_)	This function adds the data contained in *p*_*b*_ to the STM layer represented by *p*_*a*_.
*removeDataFromSTM*(*p*_*a*_, *p*_*b*_)	This function removes the data contained in the angular region *p*_*b*_ from the STM layer represented by *p*_*a*_.
*removeAllDataFromSTM*(*p*_*a*_)	This function removes all data contained in the STM layer represented by *p*_*a*_.
*createLayerInSTM*()	This function adds a new layer to the Short-Term Memory and returns a reference to it. (Layers are organized according to a LIFO rule)
*destroyLayerInSTM*()	This function destroys the currently-active layer of the Short-Term Memory—i.e. the layer which is at the top. Additionally, this function returns the layer located just below the one previously destroyed.
*getMode*()	This function returns the operating mode in which the EG strategy is at present. Only two operating modes are considered: *MtT* and *BF*. When the EG strategy is in MtT mode, the robot is moved directly to the target point. When the EG strategy is in BF mode, the robot is moved around a blocking obstacle.
*setMode*(*p*_*a*_)	This function is used to set the operating mode of the EG strategy. (The default operating mode is assumed to be *MtT*)
*computeRt*(*p*_*a*_, *p*_*b*_)	This function determines in which angular region of *p*_*a*_—*p*_*a*_ is a STM layer—the line *p*_*b*_ lies.
*isLayer*#1(*p*_*a*_)	This function returns *true* if *p*_*a*_ is the STM layer #1; otherwise, it returns *false*.

**Table 4:** A brief description of the auxiliary functions and procedures used in algorithms 1 and 2. This table is a continuation of Table 3.

Name	Description
*isAllowed*(*p*_*a*_)	This function returns *true* if the angular region *p*_*a*_ is labelled as allowed; otherwise, it returns *false*.
*isBanned*(*p*_*a*_)	This function returns *true* if the angular region *p*_*a*_ is labelled as banned; otherwise, it returns *false*.
*isFullyBanned*(*p*_*a*_)	This function returns *true* if all angular regions in *p*_*a*_—*p*_*a*_ is a STM layer—are labelled as banned; otherwise, it returns *false*.
*isInconsistent*(*p*_*a*_, *p*_*b*_)	This function returns *true* if the angular region *p*_*a*_ is labelled as banned and the data contained in *p*_*b*_ indicate that there are no obstacles in the range of directions covered by *p*_*a*_; otherwise, it returns *false*.
*getAllowedRegionClosestTo*(*p*_*a*_, *p*_*b*_, *p*_*c*_)	This function searches for the allowed-labelled angular region in *p*_*a*_—*p*_*a*_ is a STM layer—that is closest to the angular region *p*_*b*_ clockwise or counterclockwise depending on the value of *p*_*c*_.
*getBannedRegionClosestTo*(*p*_*a*_, *p*_*b*_, *p*_*c*_)	This function searches for the banned-labelled angular region in *p*_*a*_—*p*_*a*_ is a STM layer—that is closest to the angular region *p*_*b*_ clockwise or counterclockwise depending on the value of *p*_*c*_.

## Results

This section is structured into three main blocks: in the first block, we evaluate the ability of the EG strategy to solve complex navigation problems through a set of simulated experiments; in the second block, we compare the performance of the TGF, T^2^ and EG strategies while navigation takes place within several simulated environments of gradually increasing difficulty; and, finally, in the third block, we move from simulated to real experiments.

Next, we present and briefly discuss the results of the experiments performed in each of the above-mentioned blocks.

### Blocks 1 and 2: Simulated experiments

In blocks 1 and 2, all the experiments have been conducted using the so-called *MobileSim* simulator, which is a free software developed by the Omron Adept MobileRobots company. MobileSim currently supports many models of robots. Among these models, we have chosen a *Pioneer 3-AT* platform (we have taken this decision with the aim of testing the EG strategy on a robot different to the ones employed in block 3). A Pioneer 3-AT robot is essentially equipped with four wheels—each with one motor for both steering and moving—, sixteen ultrasonic sensors—eight forward-facing and eight rear-facing—, and a SICK laser scanner. It is worth noting that, in all our simulations, the detection of obstacles has been exclusively carried out by means of the Pioneer 3-AT’s laser scanner. Besides, this laser has been configured to provide a maximum detection range of—solely—2.0 meters as well as a 180-degree viewing angle with an angular resolution of 1 degree. The reason why we have restricted so much the maximum detection range of the laser scanner is just to face the navigation strategies against more challenging conditions. In the forthcoming experiments, the size of the obstacles will be far bigger than 2.0 meters. This means that these big obstacles will only be partially detected by the laser scanner; or, in other words, the EG strategy will have to take obstacle-avoidance decisions without fully knowing the shape of the obstacles.

#### Regarding block 1

**Fig 14:**
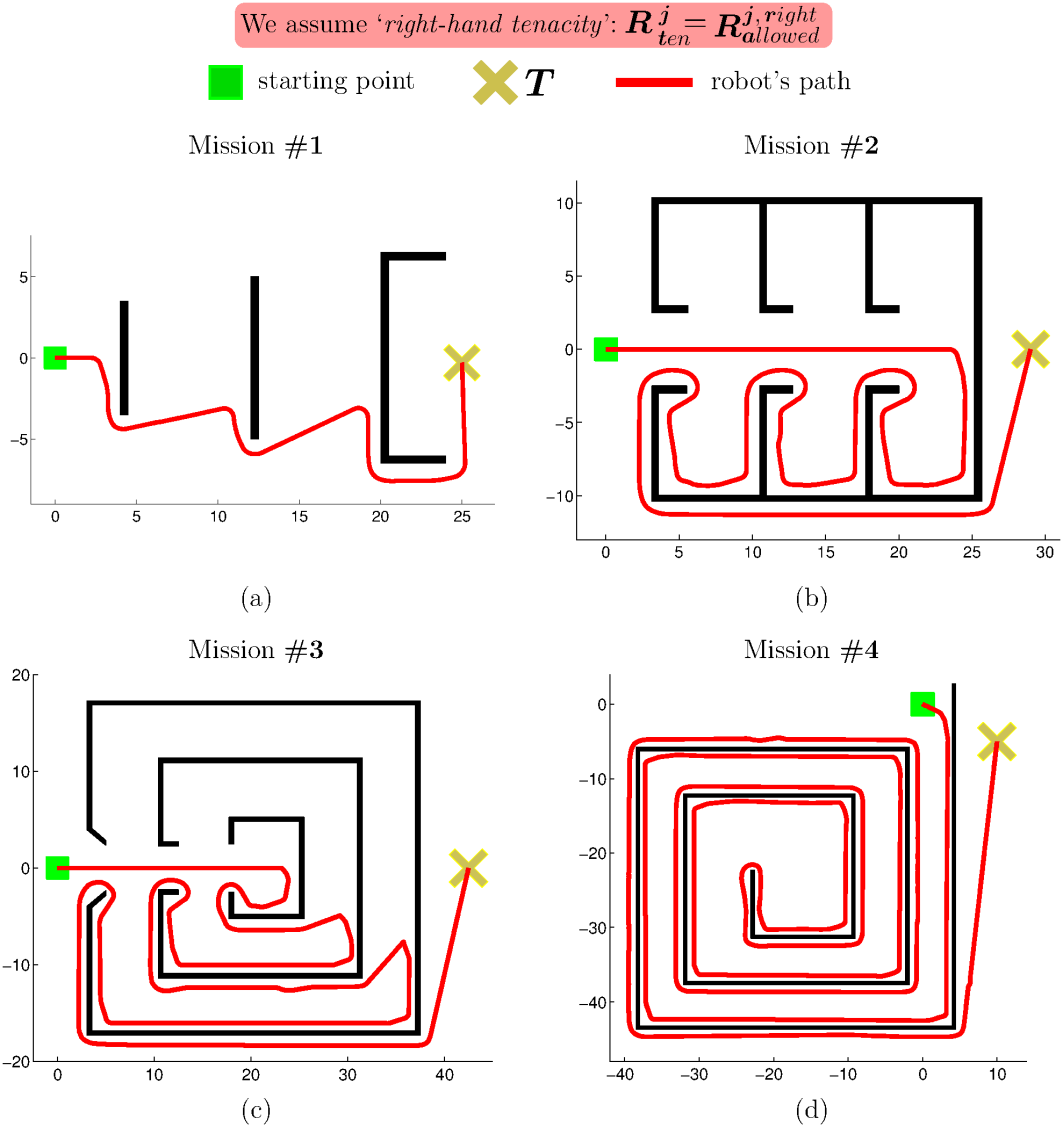
Test of the EG strategy in several scenarios where reactively-controlled robots get typically trapped when attempting to reach the target point.

**Fig 15:**
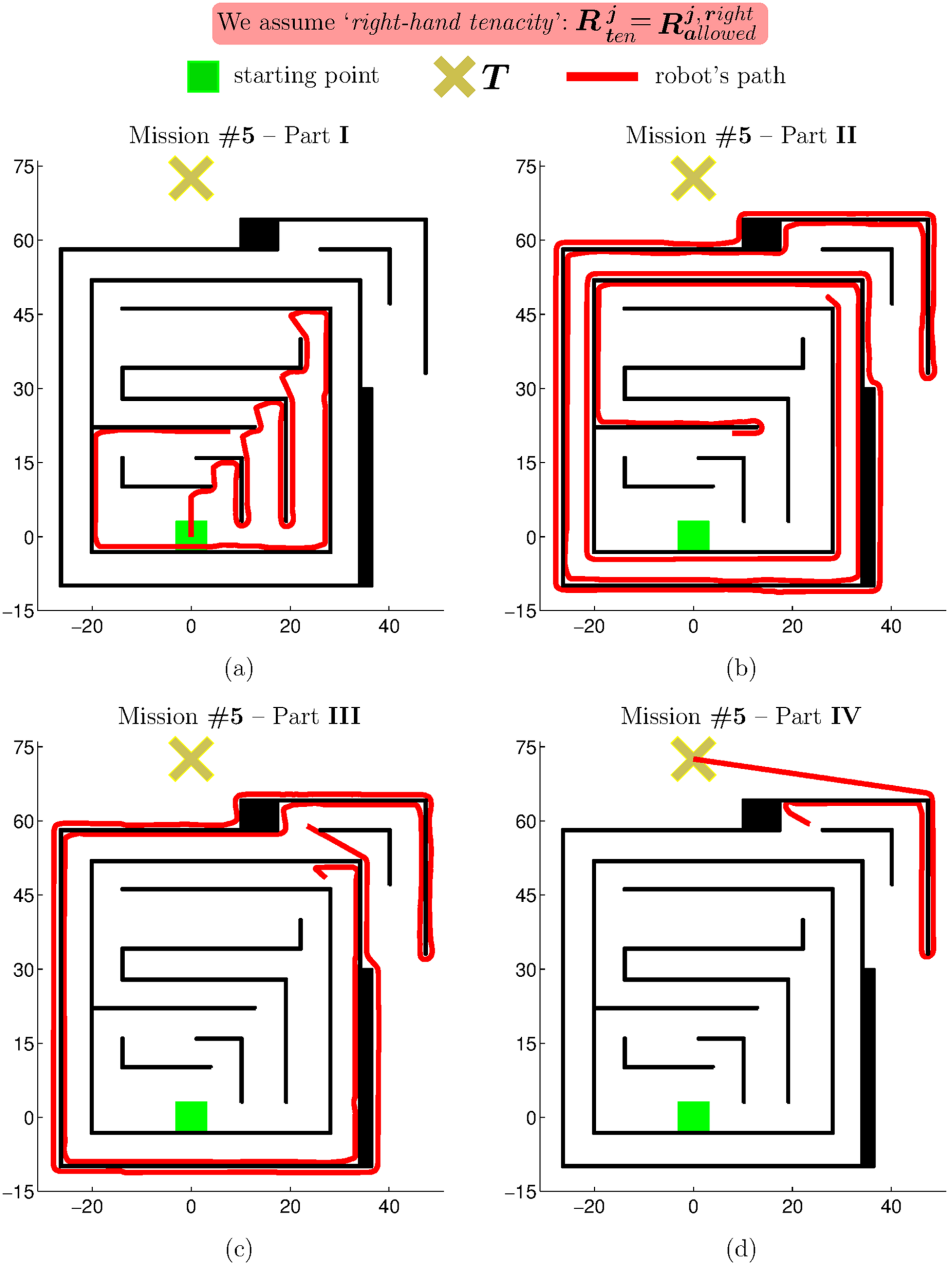
Test of the EG strategy in a scenario with a double-loop spiral-shaped obstacle. The path followed by the robot has been divided into four parts to make such a path clearer by avoiding any overlap.

**Fig 16:**
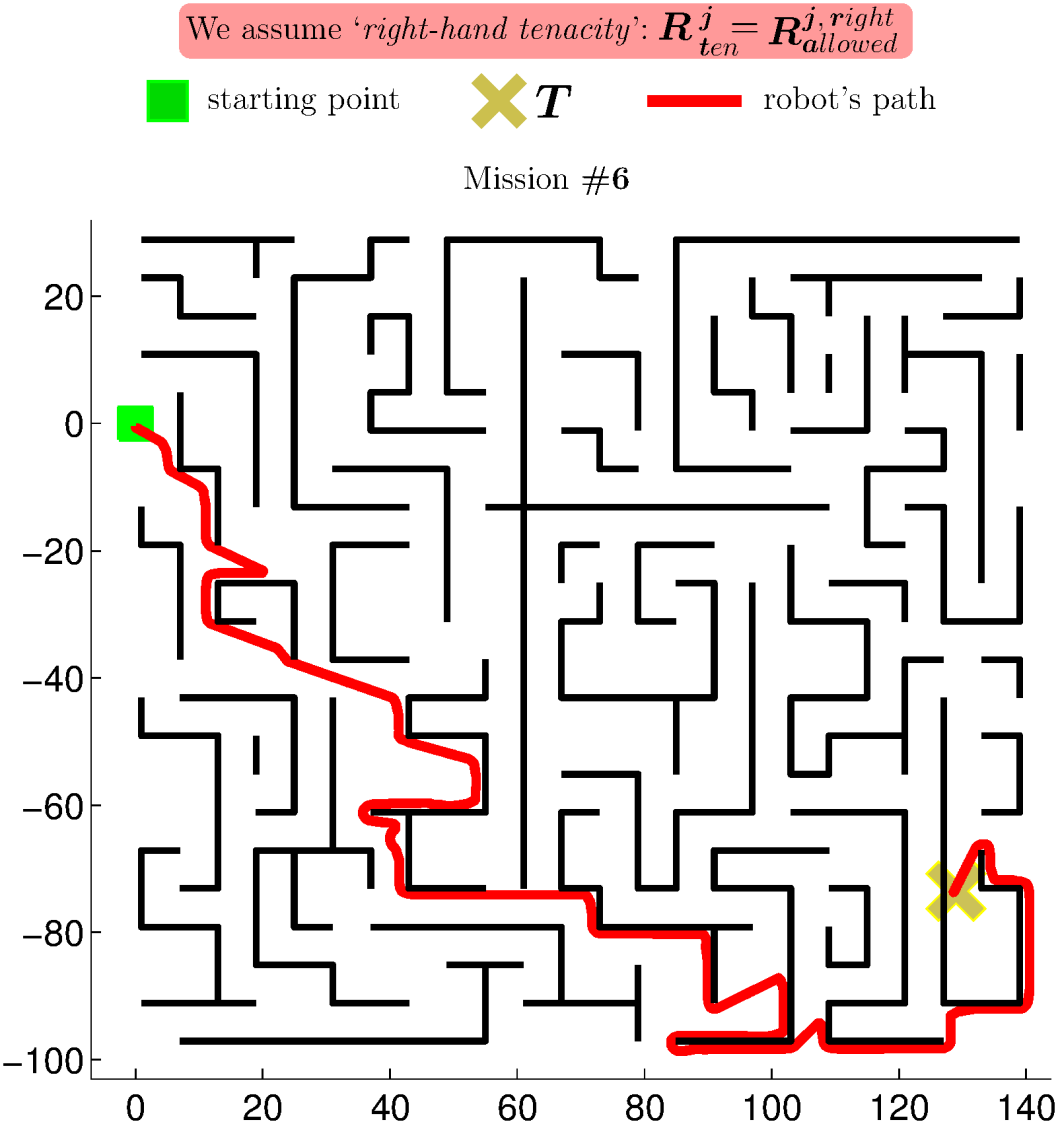
Test of the EG strategy in a large-scale scenario containing more than sixty obstacles which jointly give rise to a large maze-like obstacle.

This first block consists of the six experiments/missions depicted in Figs 14, 15 and 16 (in the plots of these three figures, the x- and y-axis units are in meters). These missions have been specifically designed to make a reactively-controlled robot fall in a trap situation.

Along the following lines, we briefly talk about the difficulties that there are behind each of the six missions included in block 1:

- In mission #1, the robot has to avoid three long walls of increasing size to get to the target point *T*.
- In mission #2, the robot has to circumnavigate a very deep box-shaped canyon.
- Mission #3 can be thought of as an extension of mission #2. Now, the robot has to escape from three box-shaped canyons of increasing size.
- In missions #4 and #5, the robot has to navigate along the boundary of an obstacle with a spiral-like shape. In mission #4, the robot is initially outside the spiral-shaped obstacle. In mission #5, the robot is initially located in the innermost loop.
- In mission #6, the robot has to find a path to *T* in a maze.

By observing the path followed by the robot in Figs 14, 15 and 16 (the red line), one quickly realizes that the EG strategy has been able to successfully drive the Pioneer 3-AT robot to the target point in all the missions which have been proposed.

#### Regarding block 2

The purpose of this second block is to compare the performance of the TGF, T^2^ and EG strategies in two different environments.

**Fig 17:**
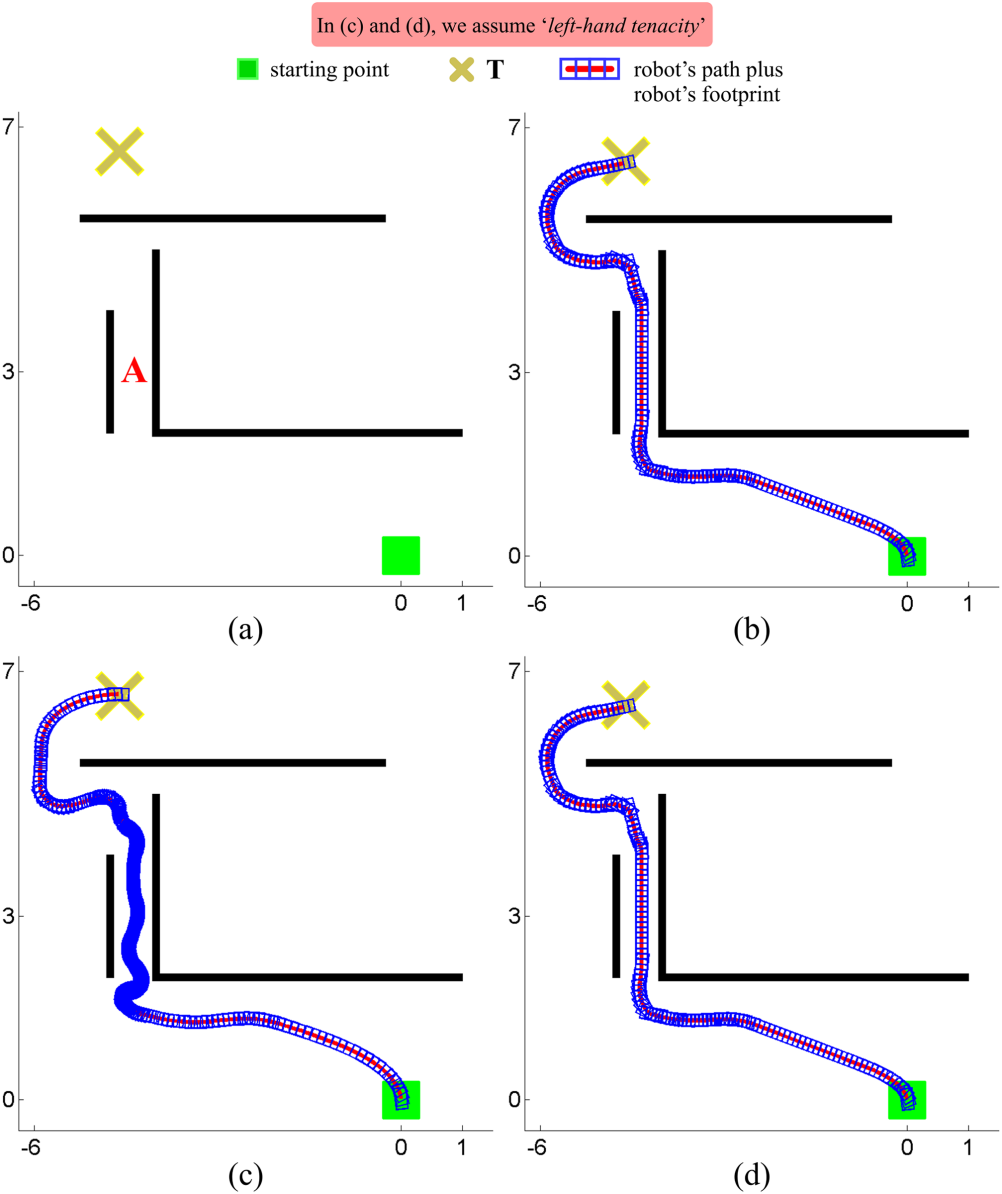
Comparing the performance of the TGF, T^2^ and EG strategies in a simple environment.

The first environment we have considered is shown in Fig 17(a). As can be seen, this environment merely comprises four long straight walls. Two of these walls create a passage labelled with letter *A*. This passage is not narrow, since its width is approximately three times the size of the Pioneer 3-AT robot. Fig 17(b), 17(c) and 17(d) depict the path taken by the Pioneer 3-AT robot while navigating according to, respectively, the TGF, T^2^ and EG strategies. As can be appreciated, the three strategies led to a quite similar path. To be precise, with T^2^, the robot exhibited a more oscillating behavior while traversing the passage A. On the other hand, with TGF and EG, the robot followed a relatively smooth path with no obvious oscillations. It is also important to highlight that TGF and EG moved the robot along exactly the same path; i.e. there is no difference between Fig 17(b) and 17(d). The reason for this is essentially two-fold: firstly, despite TGF and EG perform gap selection in a different way (remember that TGF chooses the gap which is closest to *l*_*T*_, while EG chooses the gap which is closest to *l*_*ten*_), both strategies selected the same gap at each simulation step as a consequence of the simplicity of the environment; secondly, both strategies guided the robot towards each selected gap by executing the same control commands (note that, owing to the way the EG strategy has been built, EG guides the robot to a given gap just like TGF does). Finally, Table 5 contains some quantitative data regarding the quality of the paths produced by the TGF, T^2^ and EG strategies. More precisely, these data provide information about the number of simulation steps that were necessary to move the robot to the target, and about the length and smoothness of the resultant path. To compute the smoothness of a path, we apply the so-called *Spectral Arc Length* metric [32]. This metric uses the robot’s velocity profile to quantify the smoothness of the robot’s movement. As a last point, it should be noted that the less the value for this metric, the less the smoothness of the movement.

**Table 5:** A set of metrics to quantitatively evaluate the performance of the TGF, T^2^ and EG strategies for the experiment of Fig 17. All lengths are expressed in meters.

Metric	T^2^	TGF and EG
*number* *of* *simulation* *steps*	705	430
*path* *length*	12.37	11.51
*path* *smoothness* *using* *the* *linear* *velocity* *profile*	−18.65	−5.72
*path* *smoothness* *using* *the* *angular* *velocity* *profile*	−43.25	−17.05

**Fig 18:**
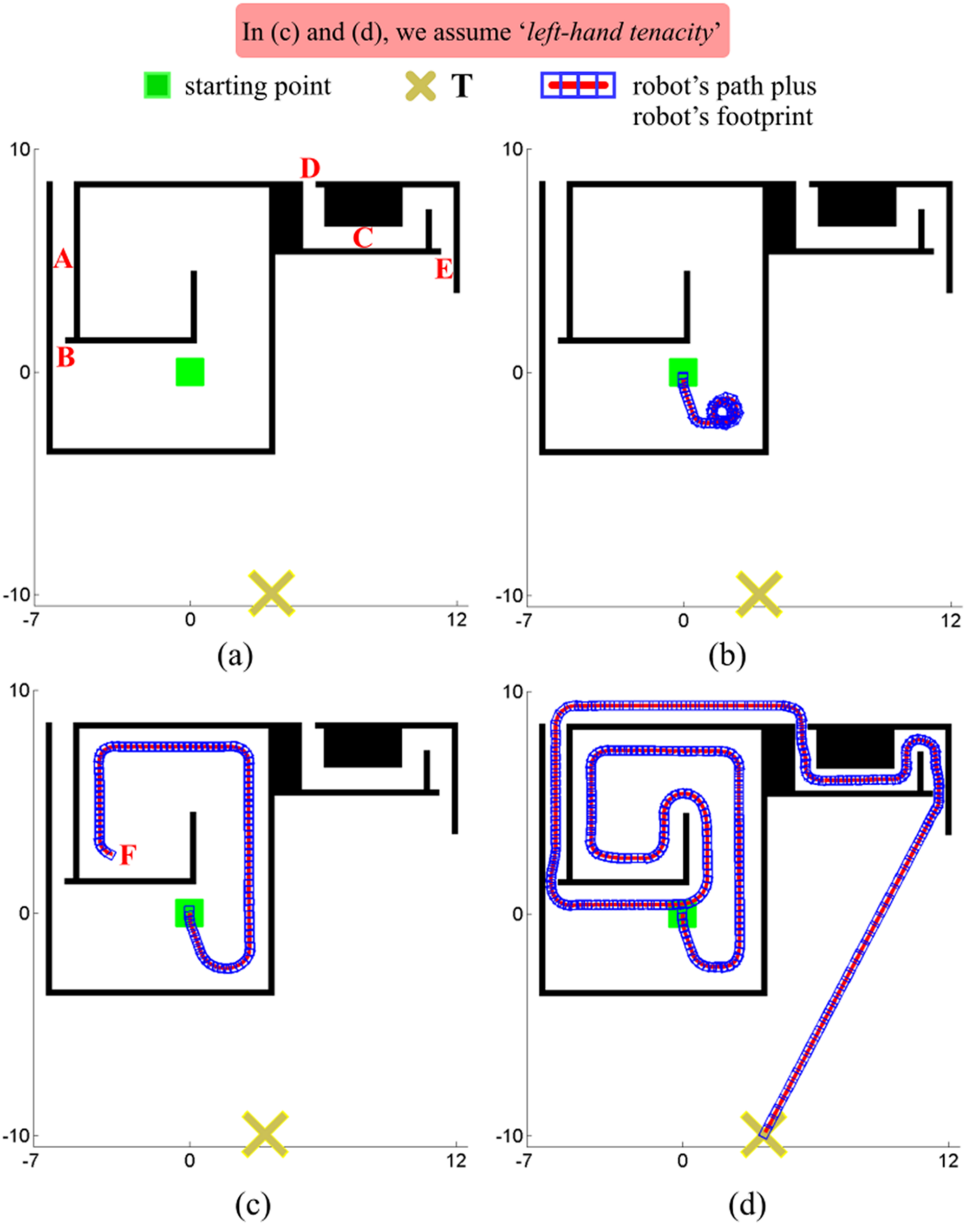
Comparing the performance of the TGF, T^2^ and EG strategies in a complex environment.

The second environment we have considered can be found in Fig 18(a). As can be observed, this environment contains a large spiral-like obstacle. To get out from this spiral, the robot is forced to pass through a long passage, which is labelled with the letter *A*. Besides, the entrance to this passage—see letter *B*—is very narrow (just a few centimeters wider than the Pioneer 3-AT’s body). Attached to the top-right side of the spiral, there is another passage—now, with a meandering shape—, which is labelled with the letter *C*. The entrance and exit points to this passage—see letters *D* and *E*, respectively—are as narrow as B. Fig 18(b), 18(c) and 18(d) show the path taken by the Pioneer 3-AT robot while navigating according to the TGF, T^2^ and EG strategies, respectively. We next discuss these results briefly. On the one hand, when the TGF strategy was used—look at Fig 18(b)—, the robot kept going round and round in circles at the bottom-right corner of the spiral-like obstacle (this result is due to the fact that the TGF strategy is generally unable to make the robot overcome an obstacle which is bigger in size than the maximum detection range of the laser scanner; in this experiment, the maximum detection range of the Pioneer 3-AT’s laser was set to 2.0 metres, whereas the size of the spiral in height and width was approximately 12×10 meters—note that, in all the plots appearing in Fig 18, the x- and y-axis units are in meters). On the other hand, with the T^2^ strategy—look at Fig 18(c)—, the robot stopped at point *F* and did not continue moving anymore (the reason for this is that the robot was at the same situation as the one of Fig 11(a), where all angular regions in the short-term memory were labelled as banned; consequently, the robot did not know where to go). Lastly, concerning the EG strategy, it successfully moved the robot to the target point, as evidenced by Fig 18(d) and S1 Video. For this experiment, we have not used metrics to quantitatively compare the performance of the TGF, T^2^ and EG strategies. In this case, we think this comparison would be unfair since the strategy which allows reaching the target point is penalized by our metrics due to the fact that it has made the robot traverse a longer path and circumnavigate more obstacles, as compared to the other two strategies for which the robot gets stuck.

### Block 3: Two real-world experiments

As a last set of experiments, the EG strategy has been implemented and tested on both a real Pioneer 3-DX robot and a real low-cost robot called Eyebot. Next, we present and briefly discuss, one by one, the results obtained in these two experiments.

#### An experiment using the Pioneer 3-DX robot

**Fig 19:**
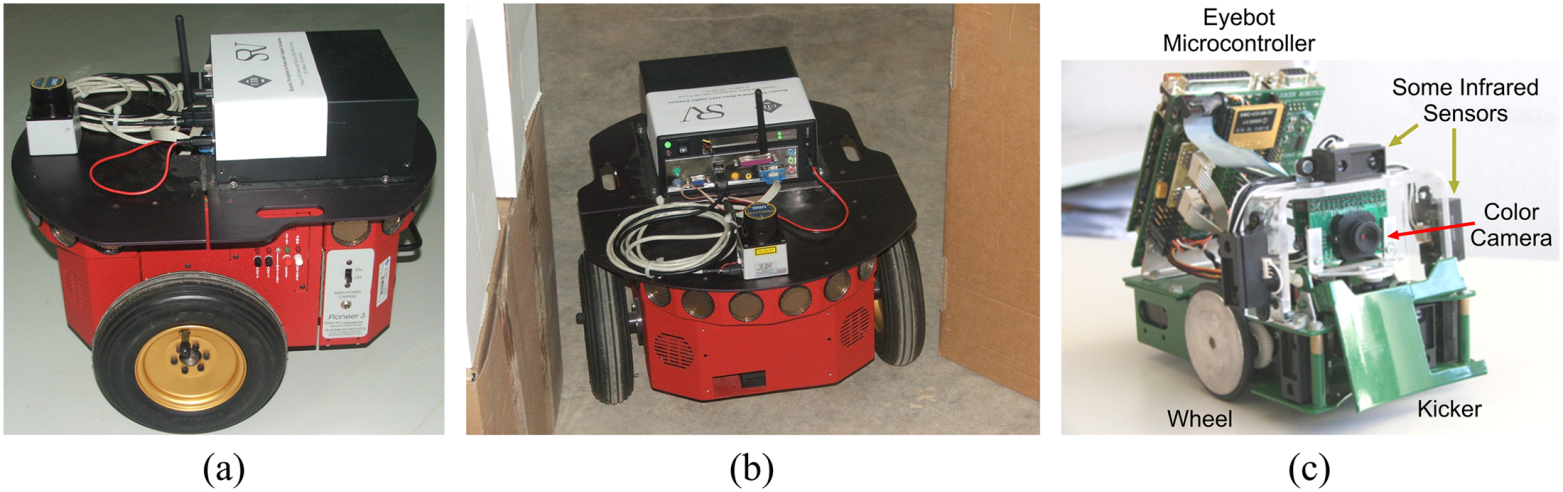
Robots used in our experiments. (a) A real Pioneer 3-DX robot. (b) A snapshot of the Pioneer 3-DX robot while performing the mission of Fig 20. (c) A real Eyebot robot.

As seen in Fig 19(a), our Pioneer 3-DX is a differential two-wheeled robot equipped with an onboard computer, a laser scanner, and sixteen ultrasonic sensors. With regard to the laser scanner, we employ a HOKUYO URG-04LX unit mounted on the front of the robot and configured for a maximum detection range of 1.7 meters as well as a 240-degree viewing angle with an angular resolution of 0.36 degrees. With regard to the ultrasonic sensors, they are arranged around the robot. In this experiment, the EG strategy has not taken into account the information provided by the ultrasonic sensors.

**Fig 20:**
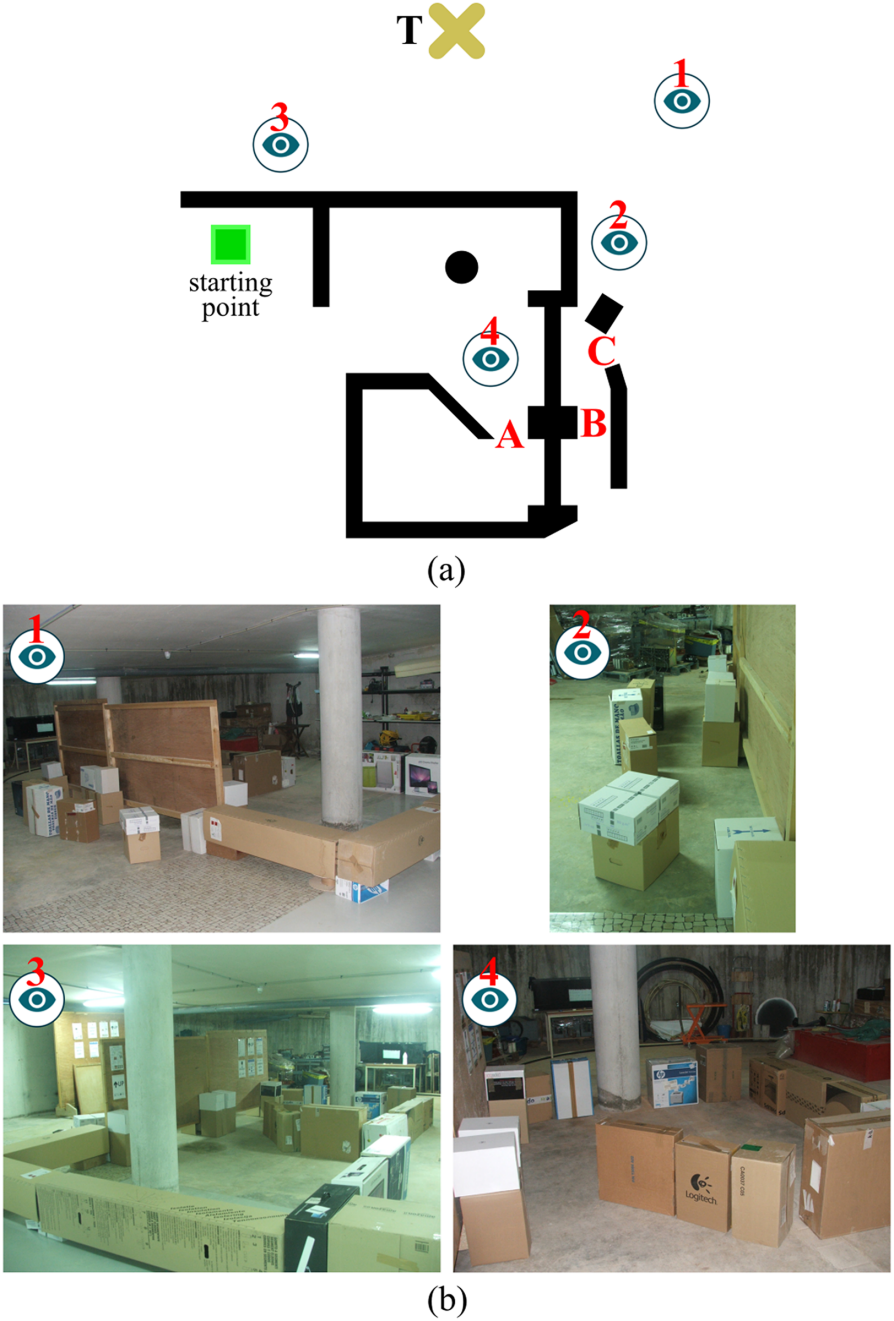
A representation of the environment where the experiment with the Pioneer 3-DX robot was conducted. (a) The environment itself (obstacles are colored in black as usual). (b) Four different views of the environment (a number has been assigned to each view; by looking for this number in Fig (a), the reader can know the approximate position from where each view was taken).

Fig 20 shows the environment where this experiment took place. As can be observed, boxes and panels have been strategically spread over the environment to form a large spiral with three narrow passages (these passages are labelled with the letters *A*, *B*, and *C* in Fig 20(a); additionally, it is important to stress that, when we say “narrow” here, we mean that the passage is just a few centimeters wider than the robot’s footprint).

**Fig 21:**
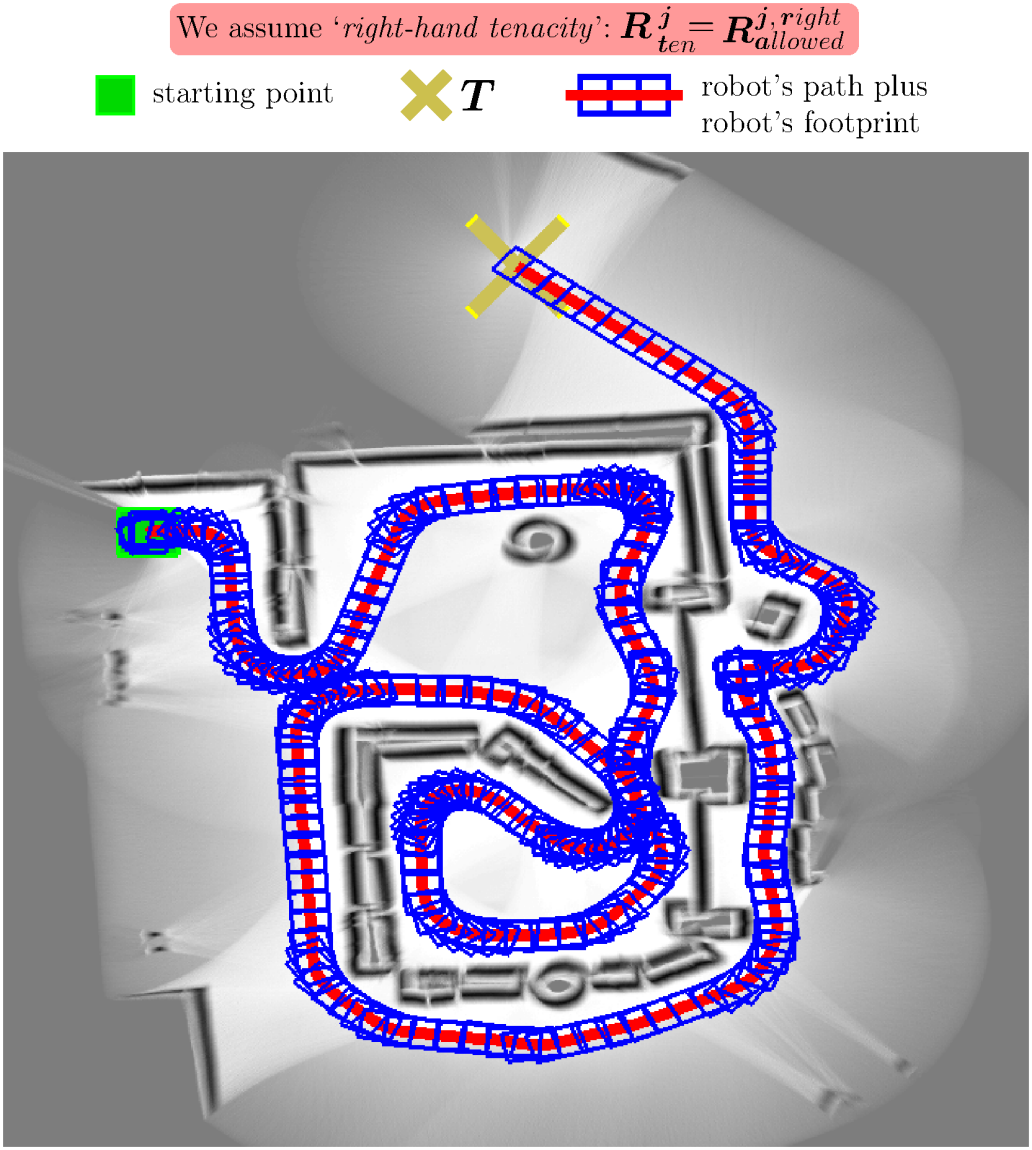
Results for the experiment with the Pioneer 3-DX robot (S2 Video). This figure shows both the path followed by the robot (over 37 meters in length), and the environment as perceived by the robot’s laser scanner.

Moving on to the results, Fig 21 plots the path—red line—that the Pioneer 3-DX robot has followed under the guidance of the EG strategy in the environment of Fig 20. From these results, we can conclude that the robot has been able to successfully attain the target point without experiencing any collision. To this end, the robot has had to circumnavigate a large spiral-shaped obstacle; what is more, the robot has had to pass through three very narrow passages (Fig 19(b) is a snapshot of the robot when it was traversing the narrow passage labelled with the letter *A* in Fig 20(a)).

#### An experiment using the Eyebot robot

Finally, a real experiment using a low-cost robot called Eyebot was carried out with a dual purpose: on the one hand, to make evident that the computational cost of the EG strategy is small enough to be run, in real time, on a microcontroller; and, on the other hand, to demonstrate that good navigation results can be obtained even with robots having cheap and inaccurate sensors for obstacle detection, such as infrared range sensors (IR).

Eyebot is a miniature mobile robot designed to meet the regulations of the RoboCup and FIRA small size leagues. Fig 19(c) shows the aspect of this robot and enumerates, at the same time, its more relevant characteristics. Among these characteristics, we highlight the following: Eyebot fits within a circle of 18 cm diameter; it has a differential drive actuator design consisting of two DC motors with encapsulated gears and encoders; it is essentially equipped with a 32-bit microcontroller that works at a clock frequency of 25 Mhz (the Eyebot’s microcontroller is a Motorola MC68332), 1 MB of RAM, and six IR sensors which are used to measure the distances to the obstacles (these sensors are distributed in groups of two among the left, front, and right sides of the robot; it is also important to point out that they offer a detection range which goes from 15 cm to 80 cm).

**Fig 22:**
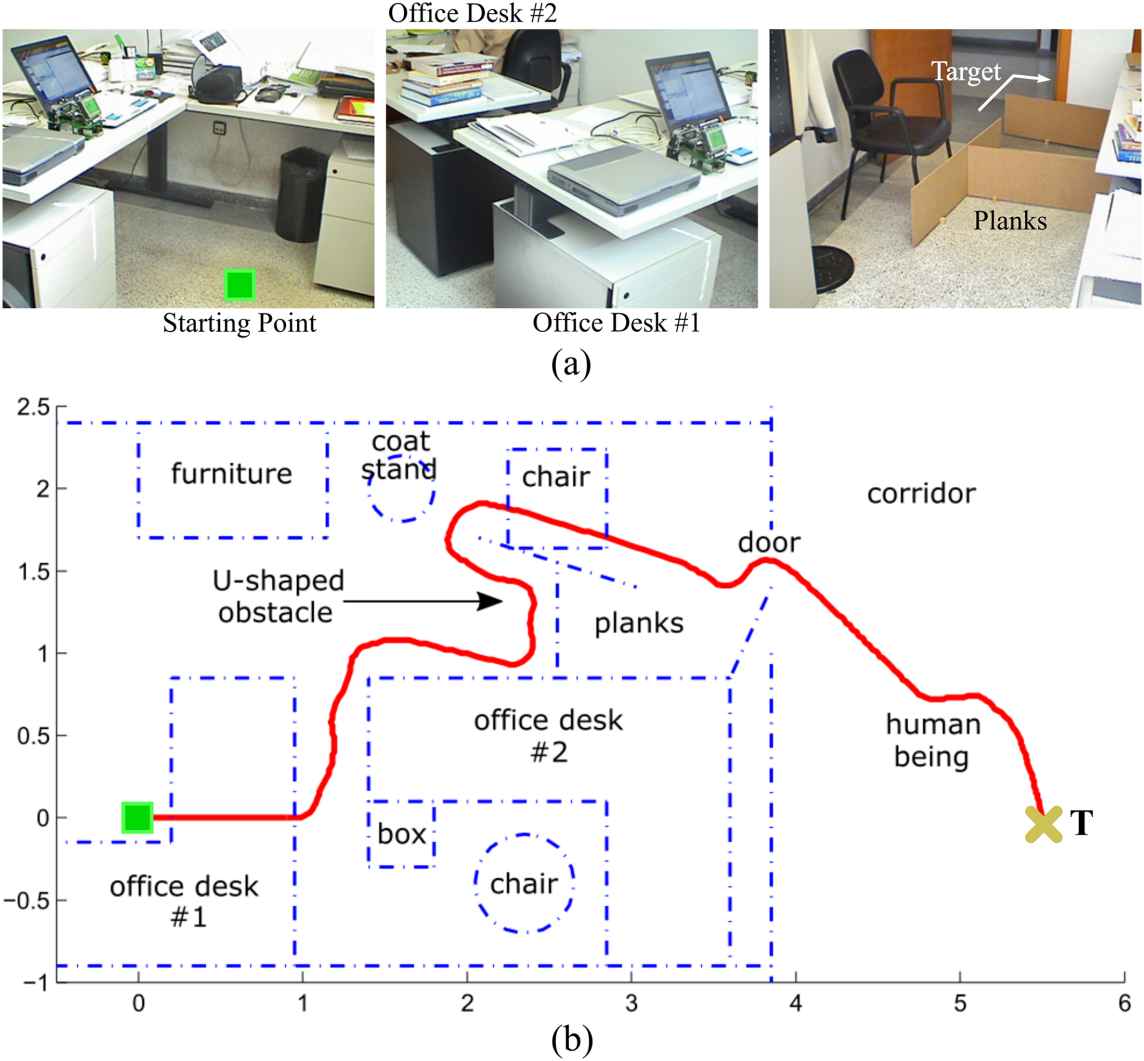
Results for the experiment with the Eyebot robot (S3 Video). In Fig (b), the x- and y-axis units are in meters.

The experiment was performed in the office-like environment outlined in Fig 22(b) (Fig 22(a) also shows some real images of the environment). The mission consisted in going from one end of the office to the main corridor. The Eyebot robot found many obstacles on its way to the target point, being, some of them, artificially created by means of planks. The resultant trajectory is drawn as a red line in Fig 22(b). As can be seen, the Eyebot robot was able to successfully reach the target without hitting any obstacle.

## Further discussion on the results

Here, we are going to discuss a series of questions that may arise after examining the results presented in the previous section (in the following, *Qx* stands for “Question number *x*”).

**Fig 23:**
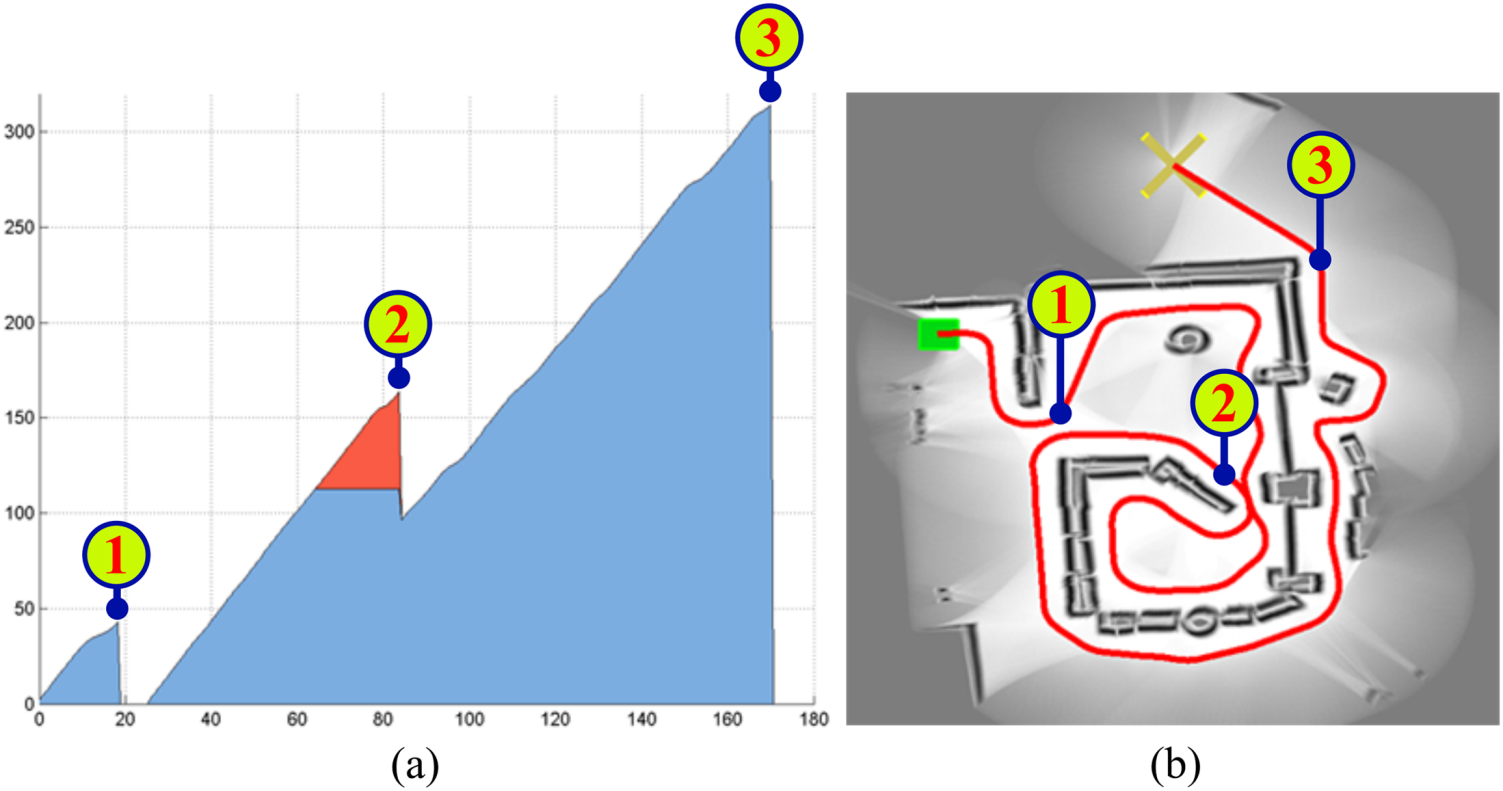
EG’s memory usage regarding the experiment of Fig 21. During this experiment, two layers were created in the short-term memory. The amount of data—expressed in KB—stored in each layer at every time instant is represented in Fig (a). This amount of data is drawn in blue color for layer #1, and in red color for layer #2. Labels 1, 2, and 3 in Fig (a) and (b) correlate memory consumption with specific points of the trajectory followed by the robot.

**Fig 24:**
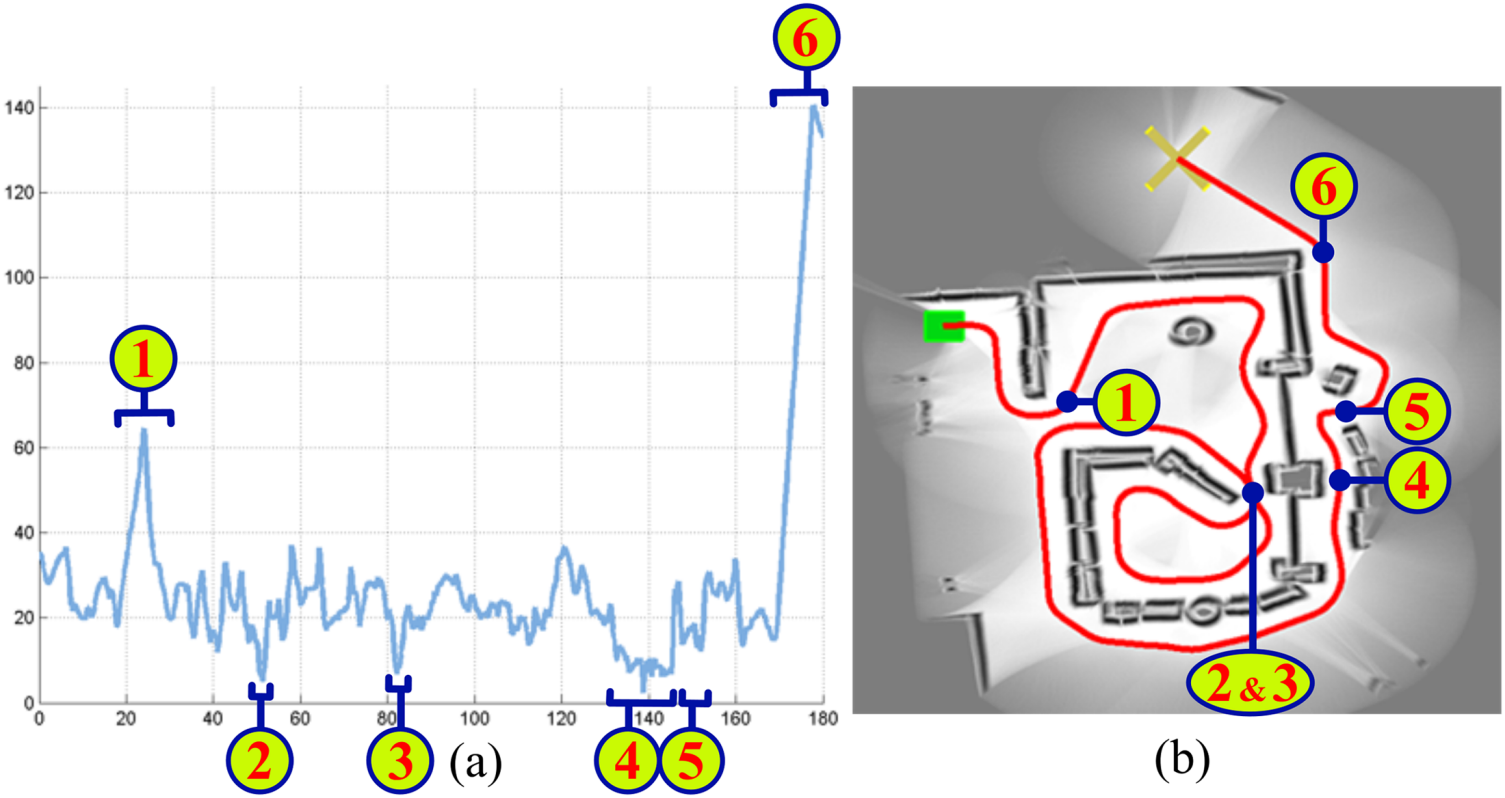
A measure of the robot’s safety regarding the experiment of Fig 21. Labels 1 to 6 in Fig (a) and (b) correlate the distance to the closest obstacle with specific points of the trajectory followed by the robot.

**Fig 25:**
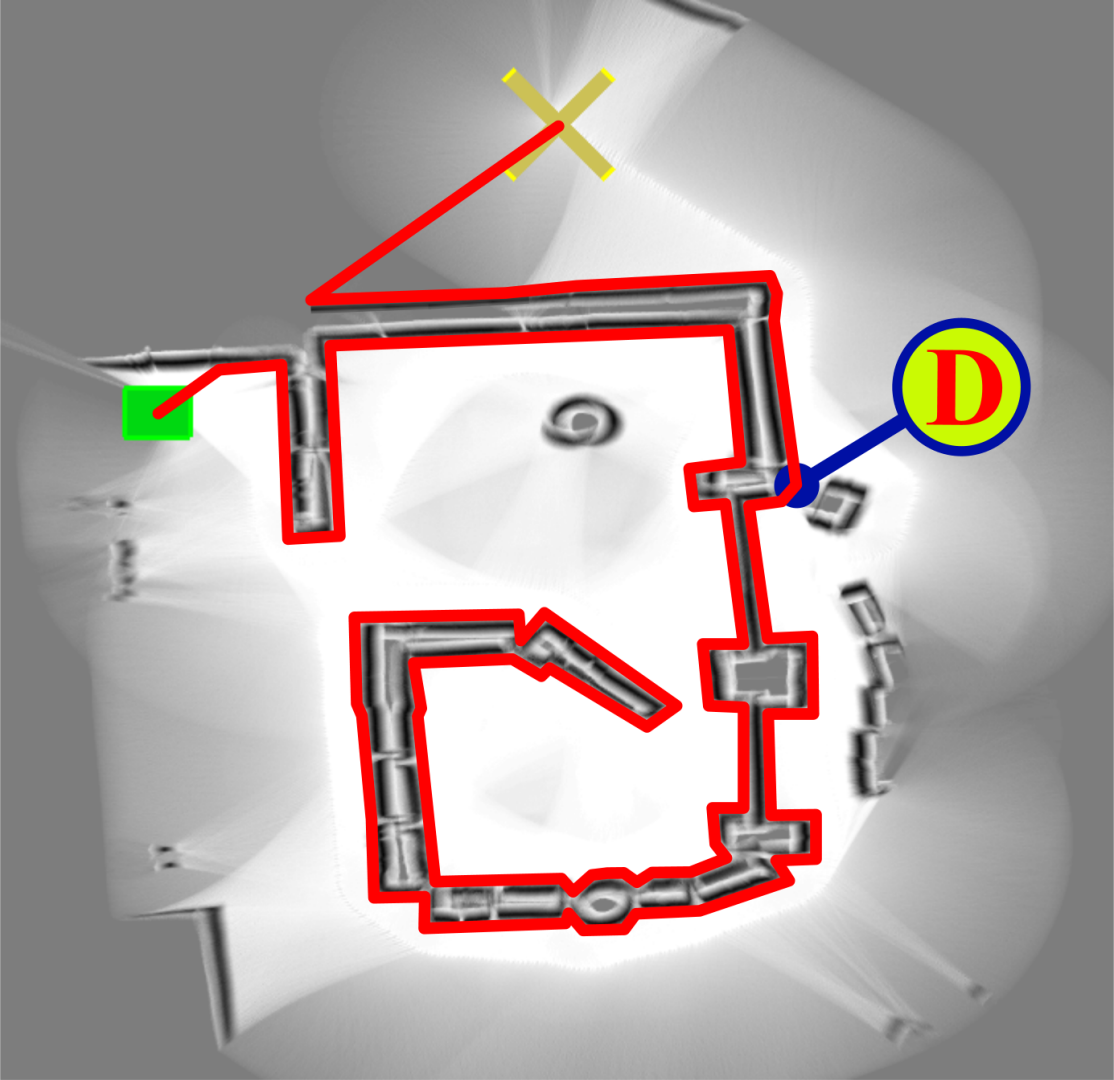
Path—in red—Generated by the Bug2 algorithm in the environment of Fig 21. Note that Bug2 was configured to follow the boundary of the obstacles to the right.

**Fig 26:**
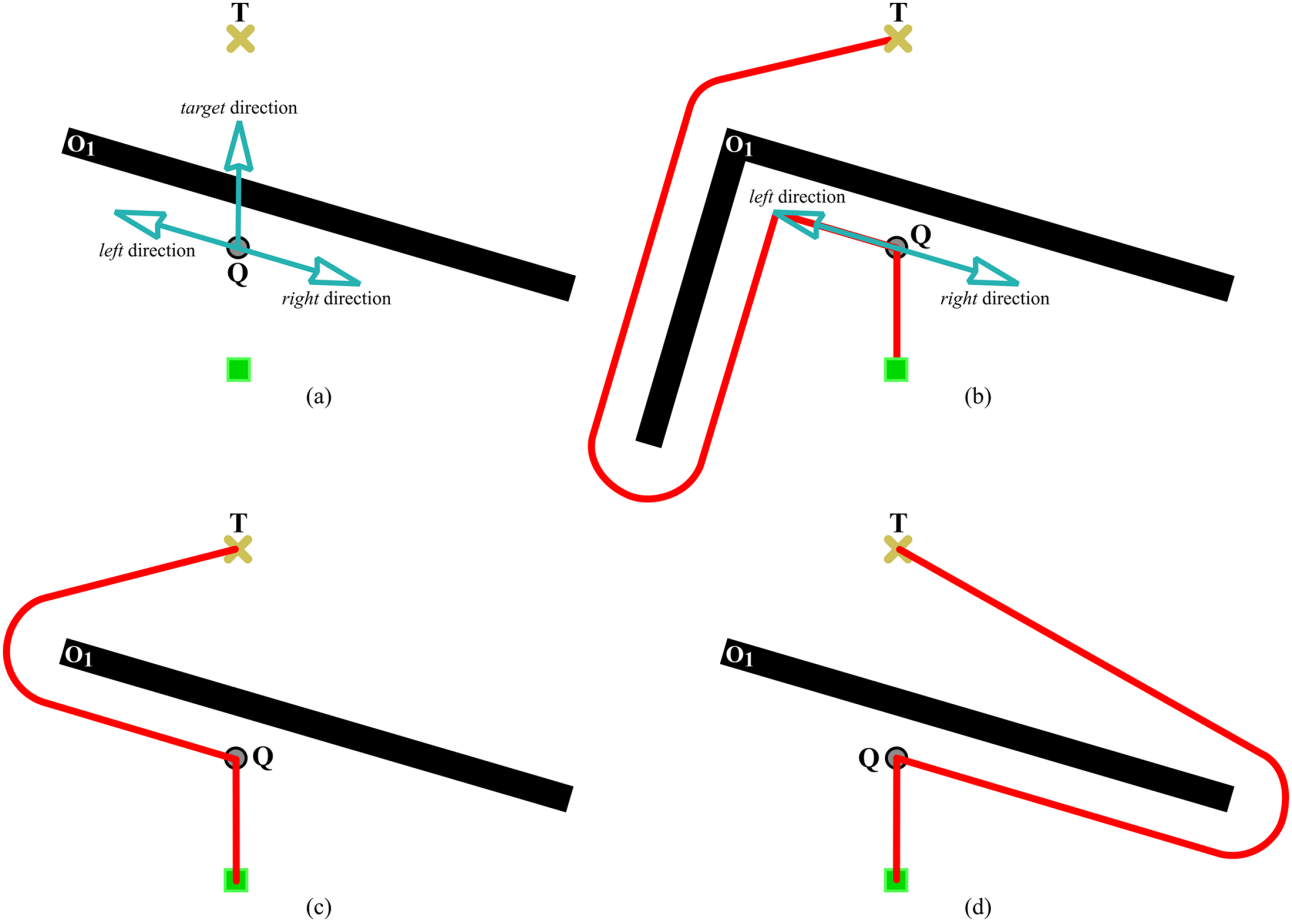
Comparing different criteria to decide in which direction the robot should circumnavigate an obstacle.

- *Q1*. The EG strategy makes use of a reasonable amount of memory while moving the robot to the desired target point. To demonstrate this, we are going to analyze the memory consumption of the EG strategy in the experiment of Fig 21. We have chosen this case among the many and different experiments run, because it took place on a real mobile robot, and because the robot had to cover a long path to get to the target.
  Fig 23(a) plots the memory consumption of the EG strategy along the 180 seconds elapsed since the robot started to move until the robot reached the target. In this figure, the x-axis units are in seconds and the y-axis units are in KB (kilobytes). As can be seen, the memory consumption was always below 350 KB; to be more precise, the memory consumption had a maximum peak of 314 KB at second 170. It can also be observed that the memory consumption dropped sharply on some occasions; specifically, at seconds 19, 84, and 170. The reasons for that were the following: on the one hand, at seconds 19 and 170, the EG strategy emptied its short-term memory because it believed that the obstacle had been avoided and that there was a free-obstacle straight-line path to the target (note that this was not true at second 19); on the other hand, at second 84, the EG strategy destroyed a layer—layer #2—which was strictly created to allow the robot to follow the boundary of the spiral-shaped part of the obstacle.
  On the basis of the above results, we can conclude that the EG strategy is able to successfully avoid large intricate-shaped obstacles by using far less than 1 MB of RAM.
- *Q2*. The EG strategy keeps the robot at a safe distance from obstacles while navigating. To demonstrate this, we have computed the distance to the closest obstacle at each point on the robot’s path for the experiment of Fig 21 (the reason for choosing this experiment is the same as that given in *Q1*).
Fig 24(a) plots the above-mentioned distances (the x-axis units are in seconds and the y-axis units are in centimeters). Peaks and valleys labelled with numbers 1 to 6 in Fig 24(a) are essentially due to the following: peaks 1 and 6 correspond to situations where the EG strategy stops following the boundary of the obstacle and moves the robot directly towards the target; valleys 2 and 3 are caused by the robot passing through the passage labelled with letter A in Fig 20(a) (the minimum value for valley 2 is 5.1 centimeters and for valley 3 is 6.8 centimeters); valley 4 represents the moment in which the robot traverses the passage labelled with letter B in Fig 20(a) (the minimum value for this valley is 2.4 centimeters); and, finally, valley 5 occurs when the robot navigates through the passage labelled with letter C in Fig 20(a) (the minimum value for this valley is 12.2 centimeters). It is important to highlight that the extremely-low minimum values for valleys 2, 3, and 4 stem from the fact that the size of passages A and B is only slightly larger than the size of the Pioneer 3-DX robot.
  If we calculate the average, the standard deviation, and the minimum value of the distances of Fig 24(a) without taking into account the six aforementioned peaks and valleys, we obtain values of, respectively, 23.8 centimeters, 5.2 centimeters, and 12.0 centimeters. From all this, we can conclude that, when the robot is performing its boundary following behavior and is not traversing a narrow passage, the robot keeps a safe distance of more than 20 centimeters from any obstacle with a reasonably low variation over time; furthermore, in the most dangerous situation of collision—given by the minimum distance value—, the robot is more than 10 centimeters from obstacles.
- *Q3*. In the section of experimental results, we have not compared the performance of the EG strategy against any Bug-like algorithm, such as *Bug1* [19], *Bug2* [19], *Tangent Bug* [21], or many others. The reason for not doing so is because we consider that this comparison would be inappropriate due to the large number of drawbacks associated with Bug-like algorithms. As is widely known, Bug-like algorithms make several assumptions which are quite unrealistic for real robots. What is more, they do not provide a complete solution to the problem of reactive navigation. All these drawbacks are discussed next in more detail.
  - Generally speaking, Bug-like algorithms assume that the robot is a point. In practical terms, this means that the robot is supposed to pass through any passage, even when this passage is narrower than the physical size of the robot. By way of example, Fig 25 depicts the path that the Bug2 algorithm would eventually generate in the environment of Fig 21. As can be observed, the path crosses the passage labelled with letter *D*, although this passage is not wide enough to let the real Pioneer 3-DX robot pass through.
    As opposed to Bug-like algorithms, the EG strategy takes into account only the passages/gaps which are wider than the robot’s footprint.
  - Generally speaking, Bug-like algorithms assume that the robot is equipped with perfect sensors for obstacle detection.
    In contrast to Bug-like algorithms, the TGF strategy applies different techniques for reducing the inherent uncertainty of sensor data (refer to [28] for a description of all these techniques). Thanks to this, the TGF strategy is able to perform smooth maneuvers when the robot travels through narrow passages.
    Since TGF is part of the proposed EG strategy, we can affirm that EG—just like TGF—is robust to noisy sensor data.
  - Generally speaking, Bug-like algorithms do not provide a complete solution to the problem of reactive navigation. Let us consider a situation where a certain Bug-like algorithm is intended to move a robot through a passage which is just a few centimeters wider than the robot’s footprint. How does the Bug-like algorithm make the robot pass through this passage without colliding? To achieve this goal, a specific algorithm, such as ND [17], SND [29] or TGF [28], should be inevitably implemented and used.
    Unlike Bug-like algorithms, the EG strategy offers all that is needed to make a robot successfully solve complex navigation tasks in a reactive manner. More precisely, the EG strategy provides specific algorithms for (1) avoiding obstacles with intricate shapes, and (2) moving between very closely-spaced obstacles with no risk of collision. Besides, points (1) and (2) are fulfilled under the guideline of the Lyapunov theory [33], which ensures the stability of the robot’s behavior at any time during navigation (all motion commands computed by EG are proven to be stable in the Lyapunov sense; it is important to note that this feature of EG has been inherited from the TGF strategy).
- *Q4*. With respect to the performance of the *TGF*, *T^2^*, and *EG* strategies, we can generally state the following:
  - The performance of any reactive navigation strategy largely depends on how the corresponding strategy chooses the direction—either left or right—to circumnavigate the obstacles that are blocking the robot’s intended path. By way of example, Fig 26(a) shows an environment with a long wall. In such a situation, the robot will initially progress to the target by following a straight-line path. At a certain point along this path, the robot will detect the long wall, and the robot will have to decide between moving to the left or to the right. On this occasion, it is preferable for the robot to move to the left, because, in this way, the robot will be able to get to the target through a shorter path.
    The TGF and EG strategies apply a different criterion to choose the direction for circumnavigating an obstacle. More specifically, the TGF strategy makes the robot move in the direction which is closest to the target direction. Taking Fig 26(a) as an example again and assuming that the robot has detected for the first time the long wall at point Q, TGF would choose the “left”-labelled direction to go around the wall, and, as a result, TGF would produce a path similar to the one illustrated in Fig 26(c). On the other hand, in EG, the above-mentioned decision is made according to the *toTenacity* parameter of the CALCRJTEN function (see algorithm 1). Let us now assume that: the value of the toTenacity parameter has been set to LEFT, the environment is the one of Fig 26(a), and the robot is located at point *Q*. Under these circumstances, EG would choose the “left”-labelled direction to avoid the wall, and, as a result, EG would produce a path equal to the one of Fig 26(c). If, by contrast, the value of the toTenacity parameter had been set to RIGHT, EG would choose the “right”-labelled direction to circumnavigate the wall, producing thus a path similar to the one represented in Fig 26(d).
    In our opinion, it is always possible to find an environment where the criterion used for choosing the circumnavigation direction does fail. Note that, in this context, a *fail* means that the criterion has suggested the robot to avoid the obstacle in the direction with a longer path to reach the target (here are just two examples: in Fig 26(a), a fail means choosing the “right”-labelled direction; on the contrary, in Fig 26(b), a fail means choosing the “left”-labelled direction). The above fact is the main reason why we have not programmed any specific criterion into EG to decide in which direction the robot should circumnavigate an obstacle; we have preferred to add a new parameter to the strategy in order to provide control for this.
    As opposed to EG, the TGF strategy makes a robot get stuck on large concave-shaped obstacles. In environments without this kind of obstacles, the performance of the TGF strategy in terms of path length is virtually identical to the one of the EG strategy, as long as both strategies have decided to circumnavigate each blocking obstacle in the same direction.
  - Just like EG, the T^2^ strategy makes use of the aforementioned *toTenacity* parameter to take the decision about whether a blocking obstacle should be circumnavigated in either the left or right directions.
    In contrast to EG, the T^2^ strategy is not able to safely move a robot through narrow passages. Moreover, T^2^ makes a robot get stuck on spiral-shaped obstacles. In environments without neither narrow passages nor spiral-shaped obstacles, the performance of the T^2^ strategy in terms of path length is virtually identical to the one of the EG strategy, provided that both strategies have set the toTenacity parameter to the same value.
- *Q5*. Generally speaking, strategies developed under the reactive paradigm are well-suited to move a robot in environments with dynamic obstacles without colliding. The above is definitely true in the case of the pure reactive paradigm, because these strategies have no memory, which means that they make their decisions based on data that are consistent with the current reality of the environment (as was pointed out in the introduction, strategies of pure type react directly to current sensor data). In the case of the non-pure reactive paradigm, it is not so clear that these strategies can be applied to dynamic environments. The reason for this is found in the fact that these strategies incorporate a small short-term memory, which is used to temporarily retain data from the environment. All decisions are guided by the data stored in such a short-term memory. To sum up, strategies of non-pure type partially base their current decisions on past information, which may lead to undesirable robot behaviors, mainly when the robot is navigating in an environment that continuously changes.
  Contradicting what we have generally said before, we think the EG strategy, despite its non-pure nature, is quite suitable for moving a robot among dynamic obstacles. Usually, dynamic obstacles are small in size and they have a non-intricate shape. As a direct consequence, these obstacles are expected to be quickly avoided by the EG strategy; or in other words, the task of avoidance of a dynamic obstacle is expected to last a short period of time, which in turn means that the EG’s short-term memory is not expected to contain data from this obstacle collected long time ago. All the above make us realize that, in the presence of a dynamic obstacle, the EG strategy will take decisions based on information which has been very recently gathered from the environment, and, therefore, the behavior of the robot will be largely consistent with the current reality of the environment.
  Finally, it is important to recall that, each time a dynamic—or static—obstacle is successfully avoided, the EG strategy empties its short-term memory with the aim of removing past information which is no longer necessary for completing the navigation task.

## Conclusions and future work

This work has presented a novel reactive control strategy that allows a robot both to safely navigate through narrow spaces and to escape from large obstacles, even when these obstacles have a shape which makes very difficult to find a way out—i.e. even when these obstacles are intricate. This novel strategy, called *Escape Gap*—or *EG* for short—, has been obtained by properly merging two other existing strategies, namely TGF and T^2^.

TGF [28] is a reactive control strategy of pure type whose major feature is to be capable of moving a robot in tight spaces without collisions. Alternatively, T^2^ [25] is a non-purely reactive control strategy which stands out for giving robots the ability to avoid obstacles of a big size, regardless of the maximum measuring range of the sensor used for obstacle detection. According to the above, it is clear that TGF and T^2^ are two strategies which offer complementary features, and also have complementary limitations. Getting to merge these two strategies so that their features are inherited and their limitations are removed has not been an easy task. As an example of this, in our first try to bring TGF and T^2^ together (represented by Fig 8(a)), the way the T^2^ strategy operates prevented the robot from approaching to narrow passages, essentially because such a strategy banned all small gaps (refer to Fig 9(b)). In consequence, the potentiality of the TGF strategy was fully wasted. Our solution to this problem has been to improve EG by adding a new component to it, consisting in a sensor data filter (see Fig 8(b)).

Apart from solving the problems that typically arise when merging strategies which work under different principles, new features have been included in EG which not were originally neither in TGF nor in T^2^. In this respect, the EG strategy has been enhanced with a multi-layer short-term memory (look at Fig 8(d)). Thanks to that, any robot controlled by EG has gained the skill needed to avoid obstacles which have a multi-level nesting shape, as is the case of a multi-loop spiral-shaped obstacle.

Finally, the realization of a carefully selected set of simulated and real experiments has demonstrated that: (1) EG significantly outperforms TGF and T^2^; and (2) EG takes the reactive paradigm a step further, in the sense of being able to successfully solve navigation tasks of very high complexity.

As a future work, we are planning to continue improving the EG strategy. To be more precise, such improvements will mainly focus on two key aspects. On the one hand, it is important to note that EG implicitly assumes that the mobile robot is holonomic. However, this is often not the case. By adopting the solution proposed in [34], we will ensure that EG explicitly takes non-holonomic constraints into account. On the other hand, as a natural development, the EG strategy should end up being part of an advanced hybrid control system. In this way, we will achieve the goal of moving the robot to the target point along better/shorter paths—at the cost of more computation time.

## Supporting information

S1 Video The EG strategy moving a simulated Pioneer 3-AT robot in an environment with a spiral-shaped obstacle and several narrow passages. This video can be downloaded from http://srv.uib.es/EG-simulated-exp/.
(MP4)

S2 Video The EG strategy moving a real Pioneer 3-DX robot in an environment of similar complexity to the one appearing in S1 Video. This video can be downloaded from http://srv.uib.es/EG-real-exp-pioneer/.
(MP4)

S3 Video The EG strategy moving a real Eyebot robot in an office-like environment comprising a U-shaped obstacle. This video can be downloaded from http://srv.uib.es/EG-real-exp-eyebot/.
(MP4)
